# Supplementary figures and images for: Ketamine’s rapid antidepressant effects are mediated by Ca2+-permeable AMPA receptors
Source: eLife. 2023 Jun 26;12:e86022. doi: 10.7554/eLife.86022 (PMC10319435; doi:10.7554/eLife.86022)

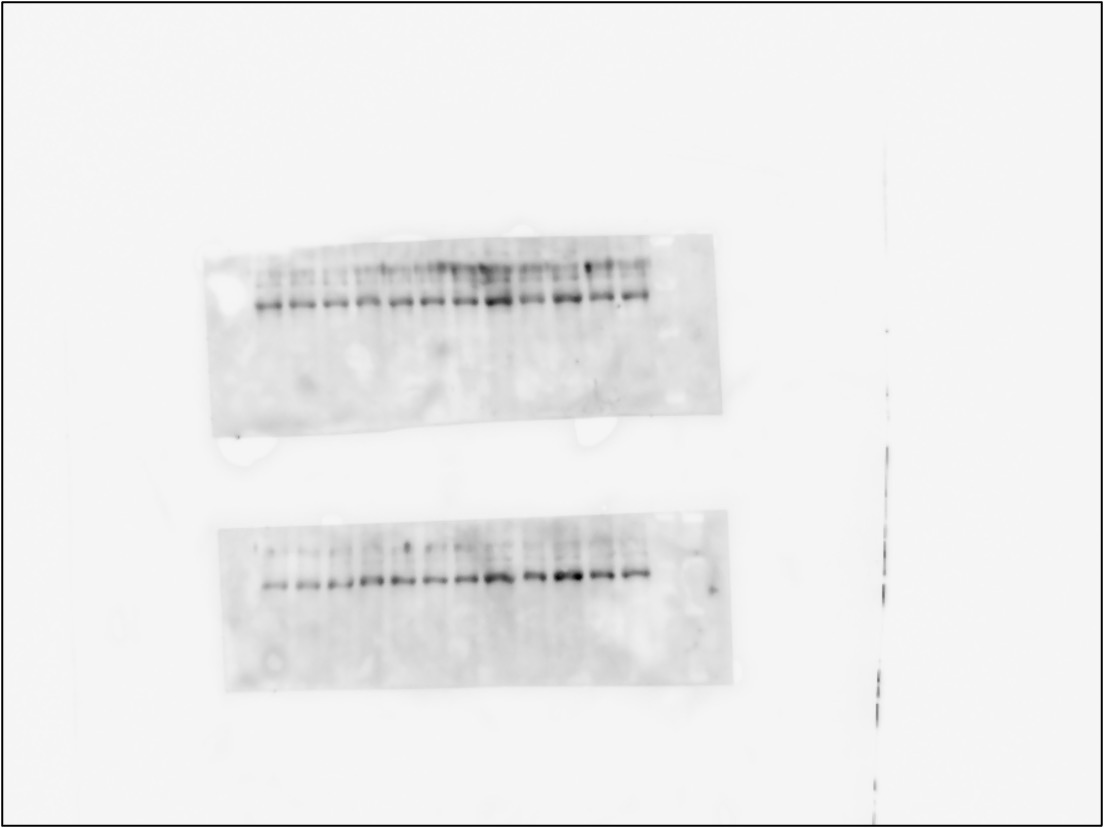

Supplement: Figure 1—source data 1. [file elife-86022-fig1-data1.zip › Figure 1 - source data 1/Figure 1 - figure supplement 3-2.jpg]

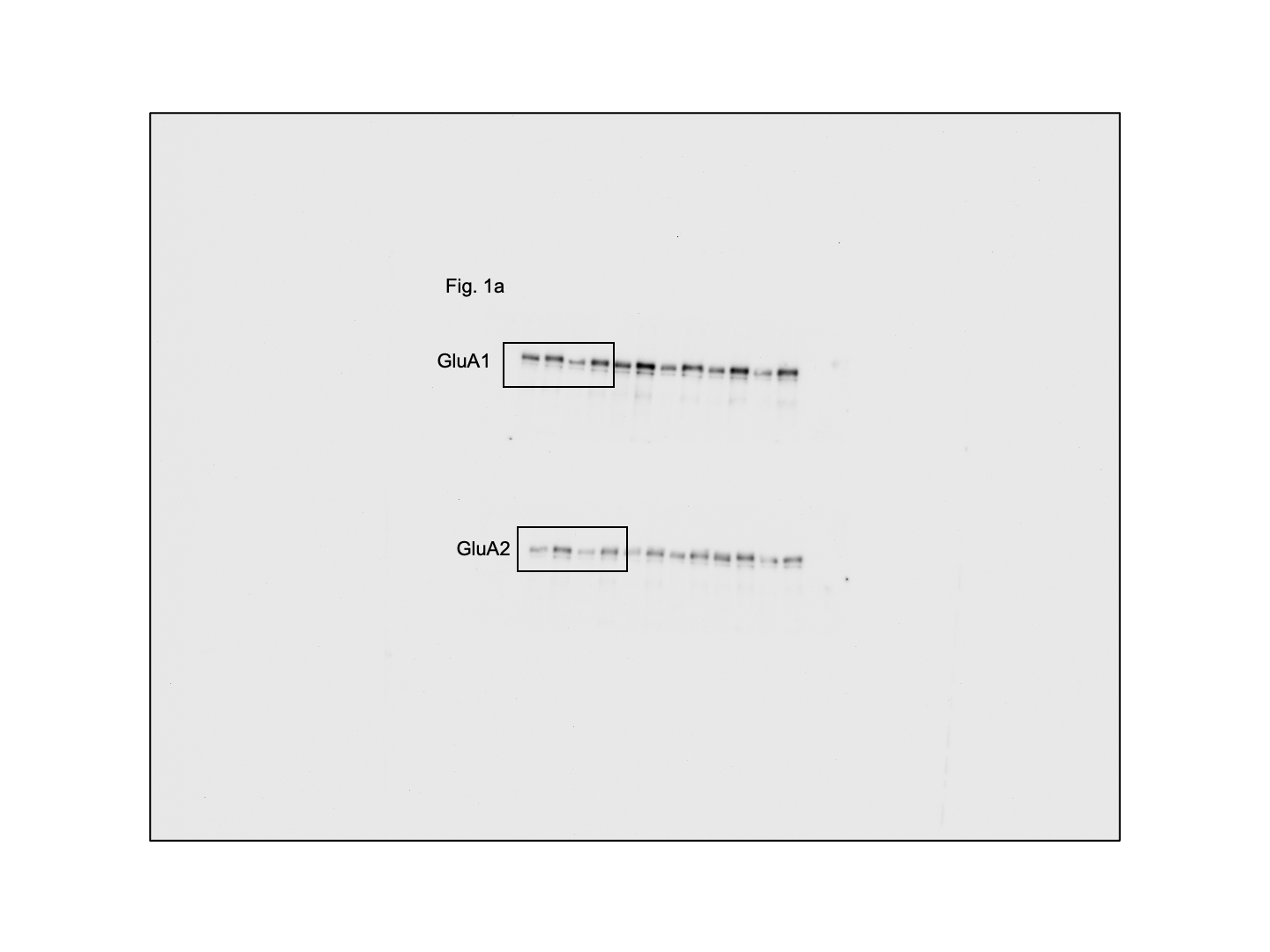

Supplement: Figure 1—source data 1. [file elife-86022-fig1-data1.zip › Figure 1 - source data 1/Figure 1 - figure supplement 1-1.tiff]

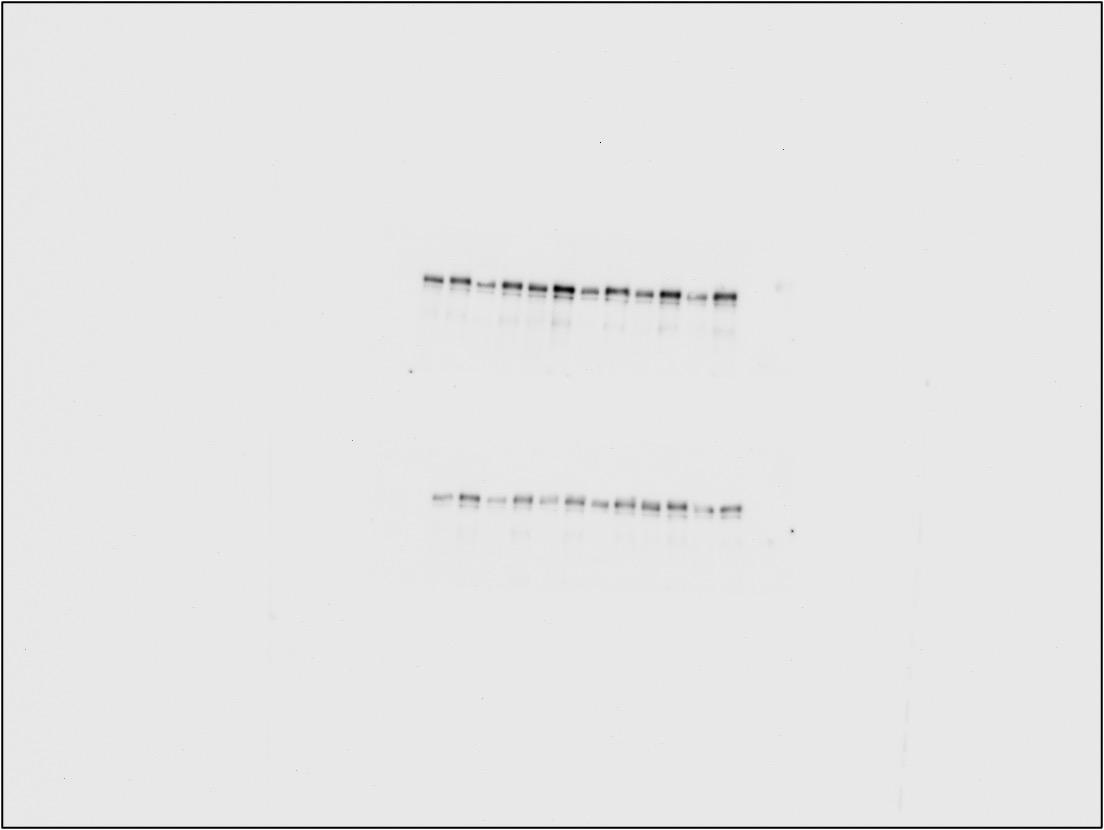

Supplement: Figure 1—source data 1. [file elife-86022-fig1-data1.zip › Figure 1 - source data 1/Figure 1 - figure supplement 1-2.jpg]

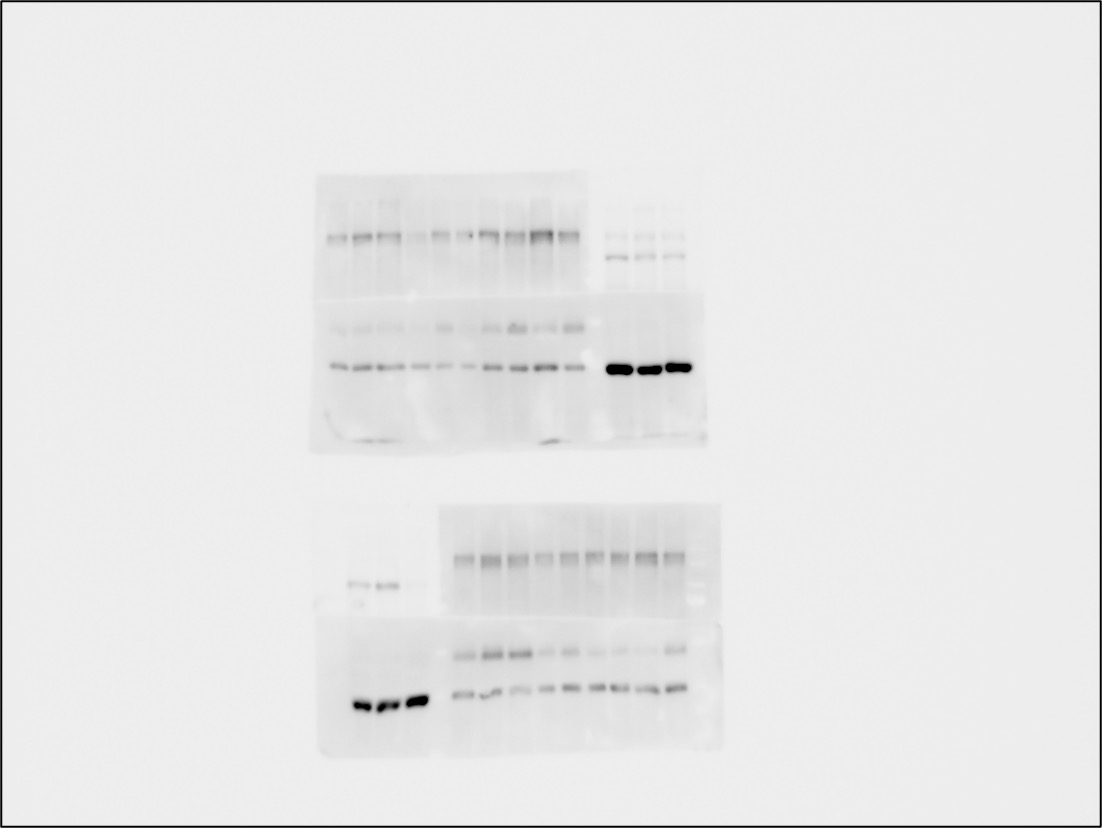

Supplement: Figure 1—source data 1. [file elife-86022-fig1-data1.zip › Figure 1 - source data 1/Figure 1 - figure supplement 5-2.jpg]

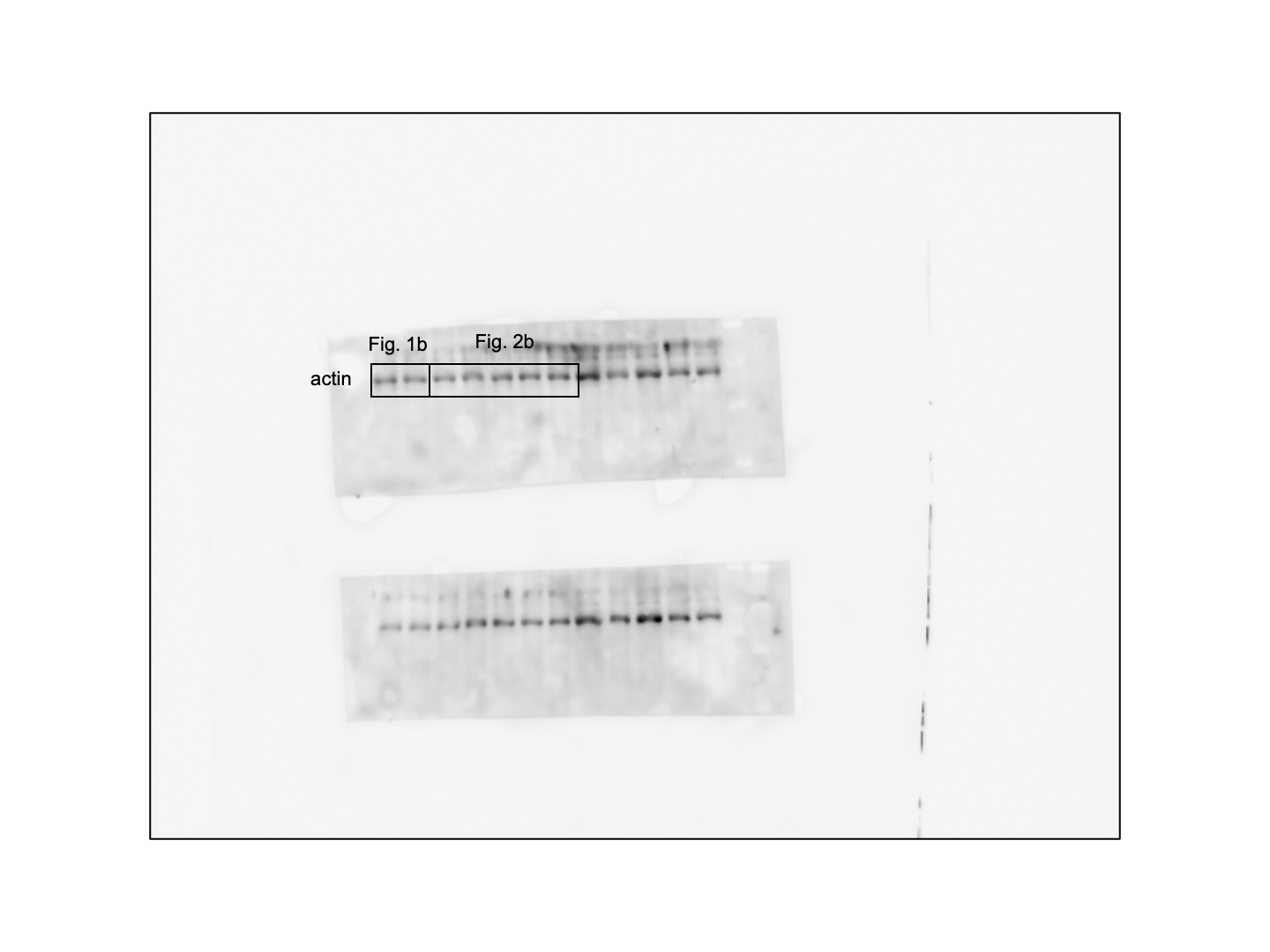

Supplement: Figure 1—source data 1. [file elife-86022-fig1-data1.zip › Figure 1 - source data 1/Figure 1 - figure supplement 3-1.tiff]

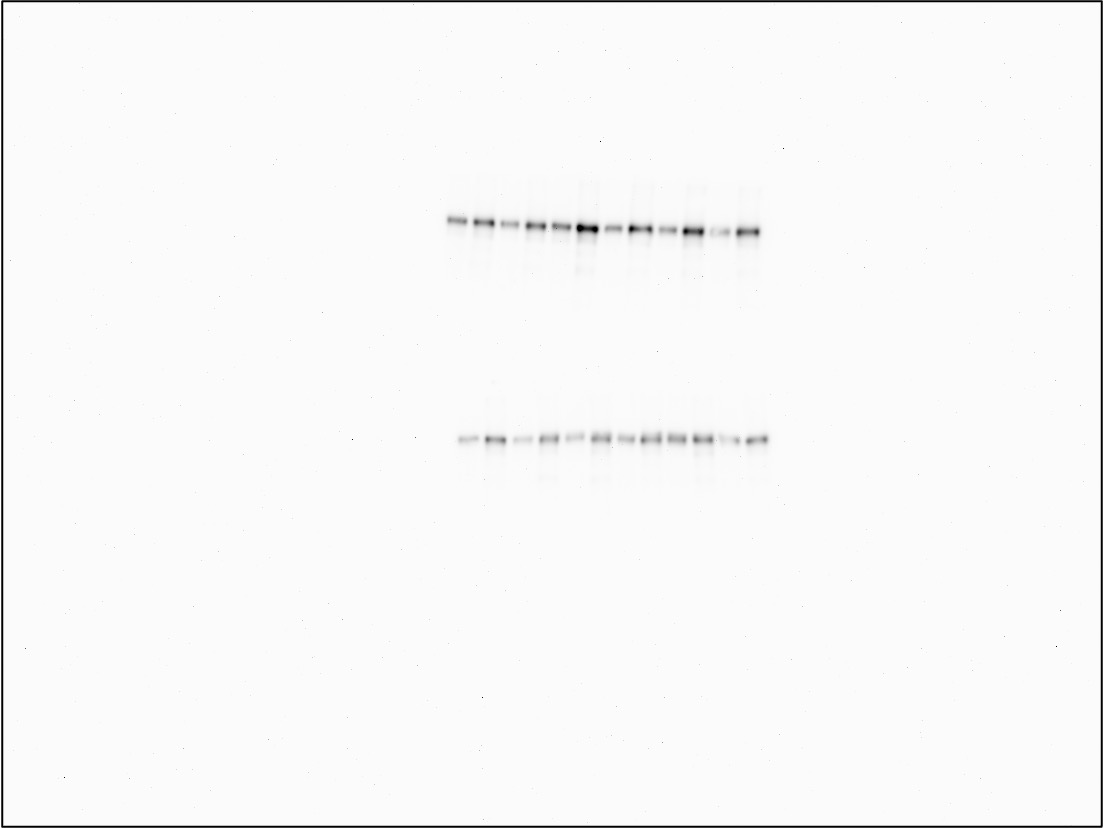

Supplement: Figure 1—source data 1. [file elife-86022-fig1-data1.zip › Figure 1 - source data 1/Figure 1 - figure supplement 2-2.jpg]

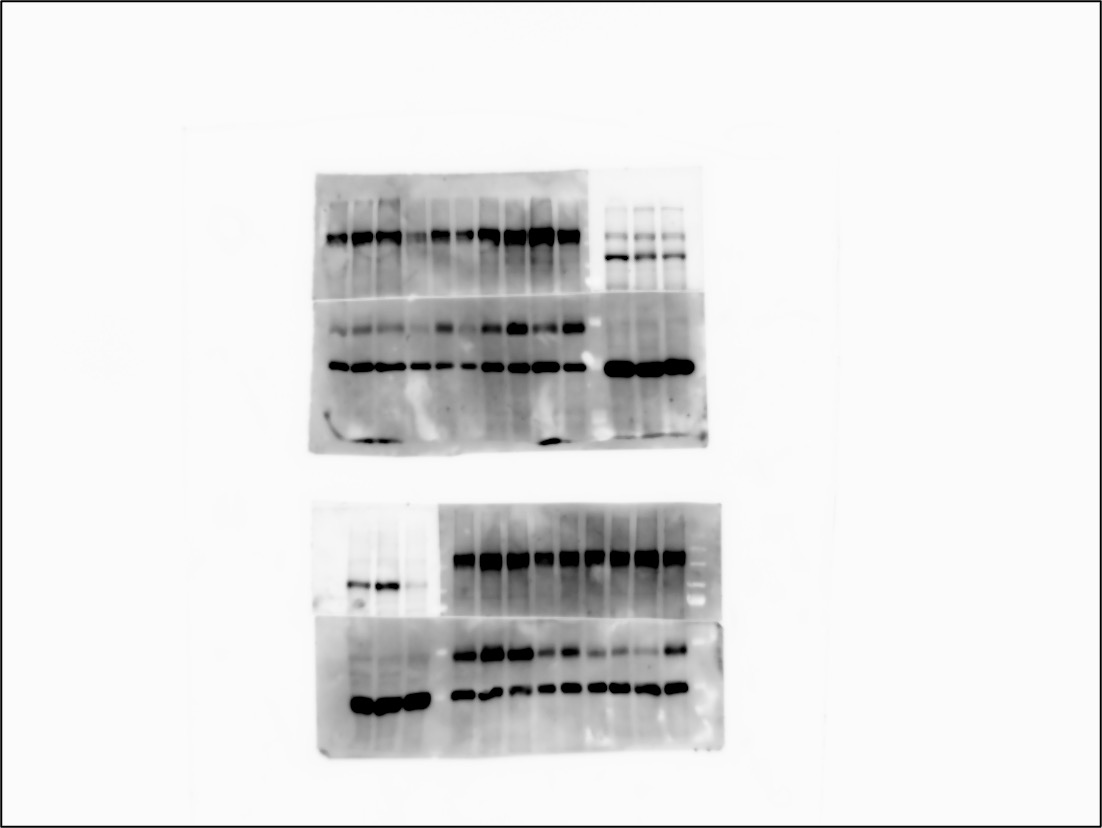

Supplement: Figure 1—source data 1. [file elife-86022-fig1-data1.zip › Figure 1 - source data 1/Figure 1 - figure supplement 4-2.jpg]

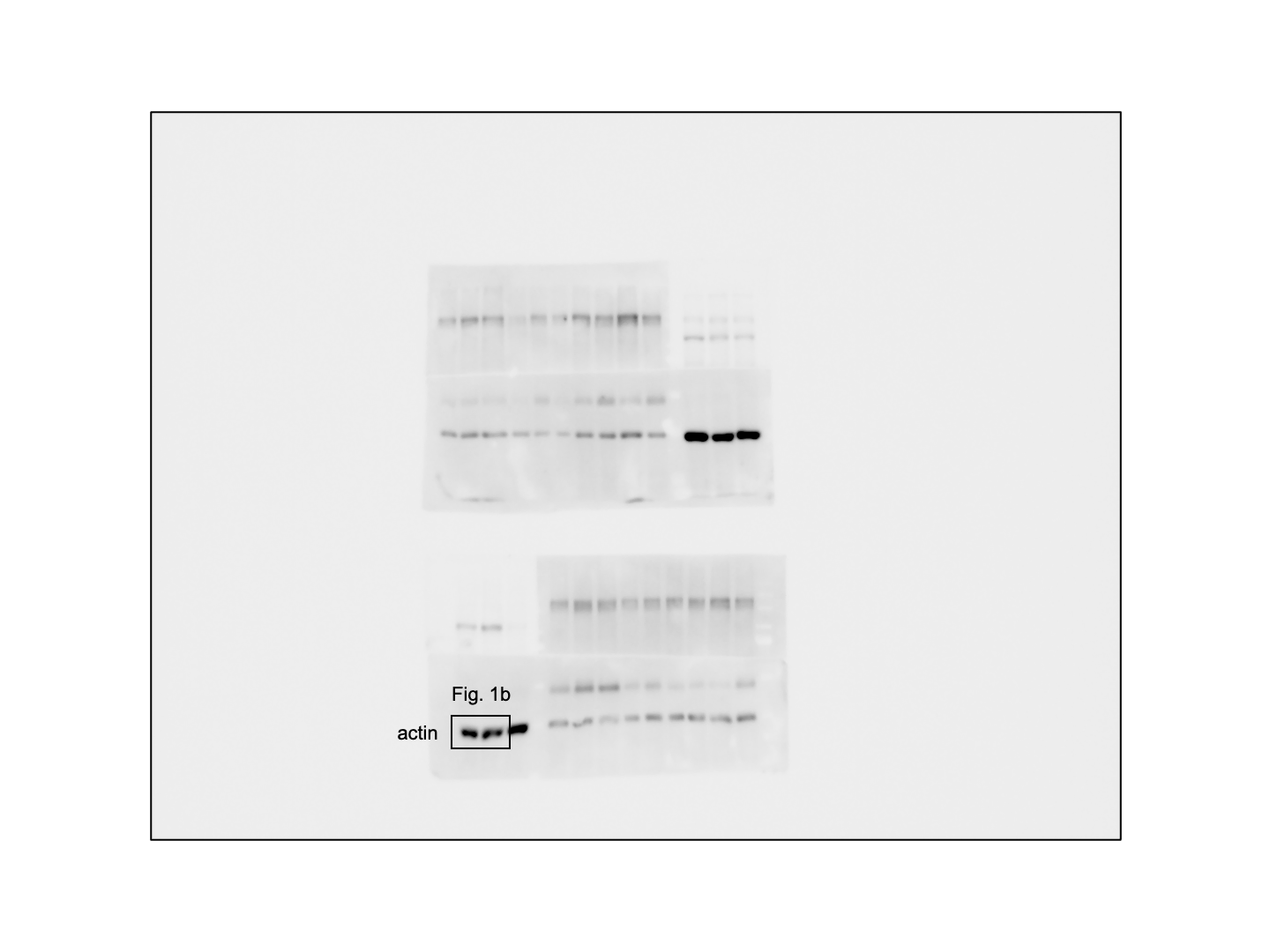

Supplement: Figure 1—source data 1. [file elife-86022-fig1-data1.zip › Figure 1 - source data 1/Figure 1 - figure supplement 5-1.tiff]

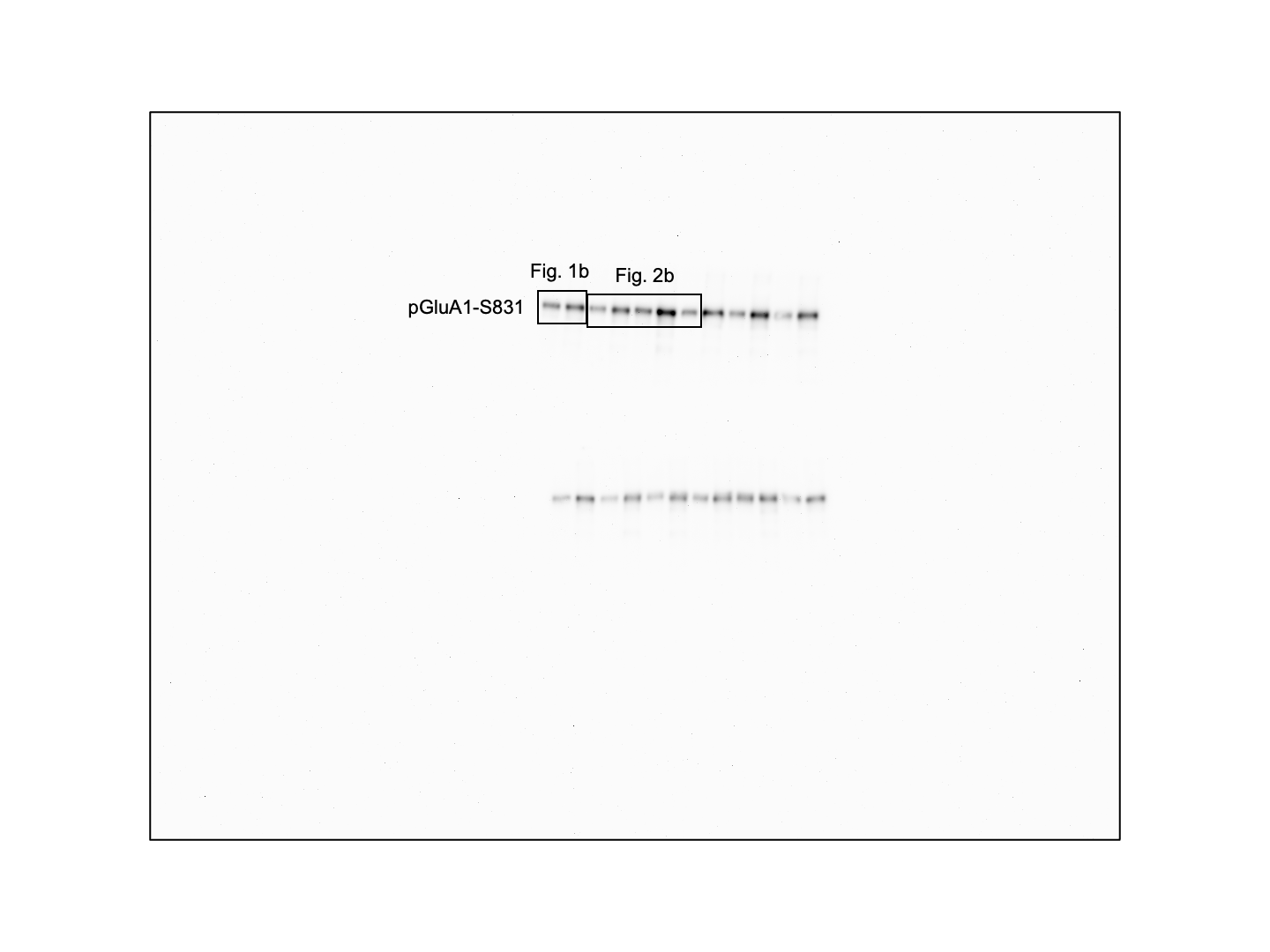

Supplement: Figure 1—source data 1. [file elife-86022-fig1-data1.zip › Figure 1 - source data 1/Figure 1 - figure supplement 2-1.tiff]

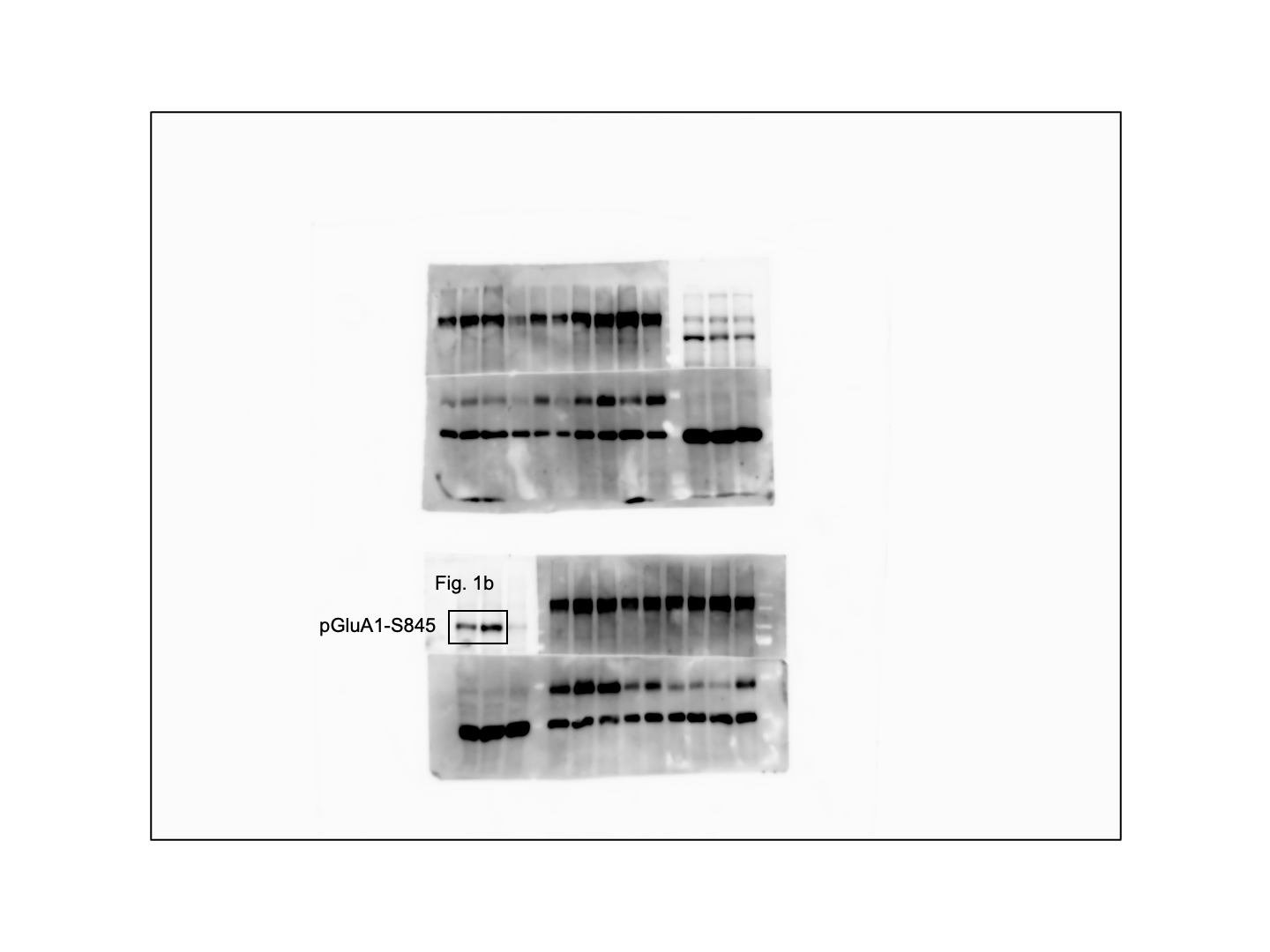

Supplement: Figure 1—source data 1. [file elife-86022-fig1-data1.zip › Figure 1 - source data 1/Figure 1 - figure supplement 4-1.tiff]

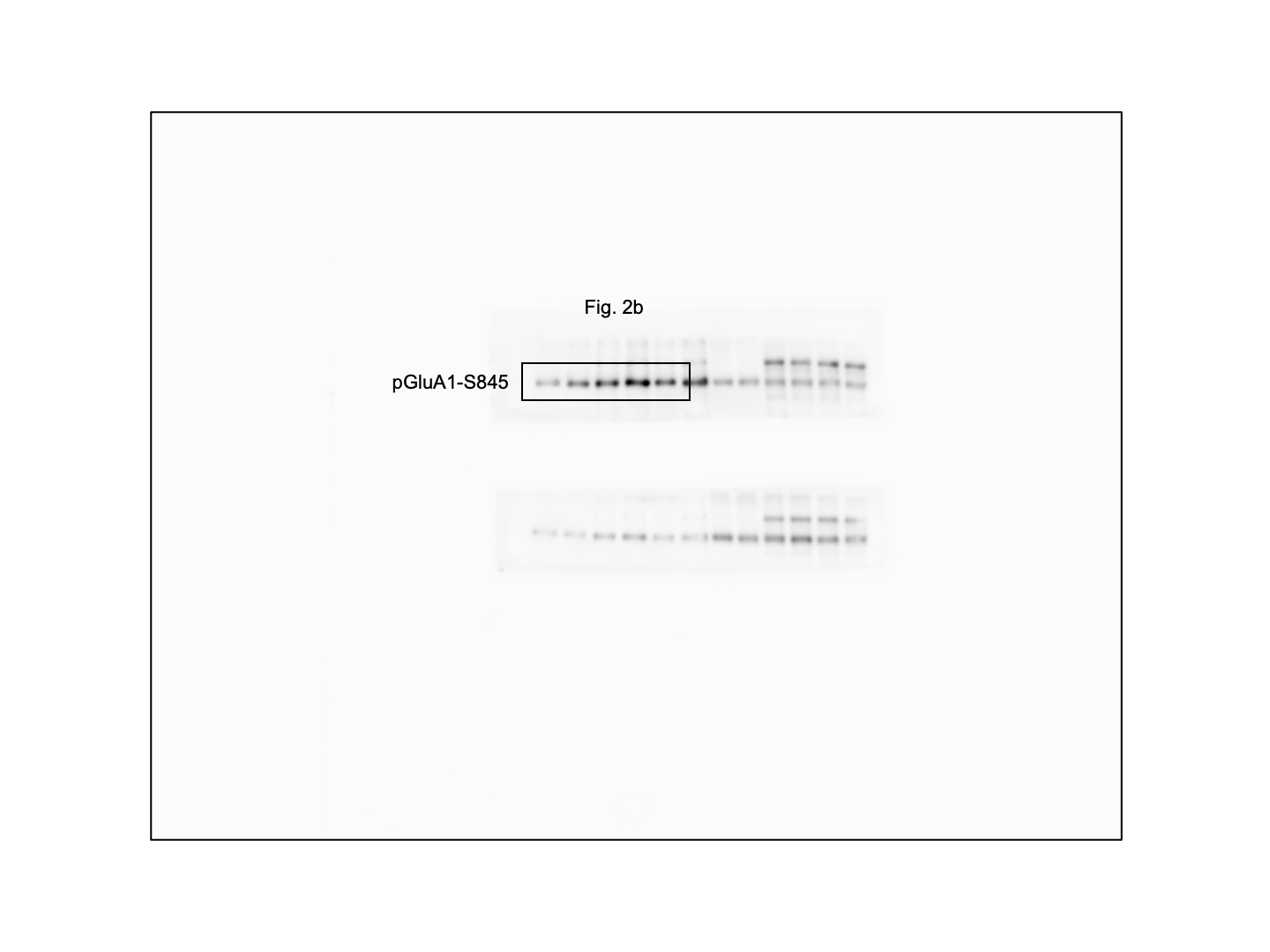

Supplement: Figure 2—source data 1. [file elife-86022-fig2-data1.zip › Figure 2 - source data 1/Figure 2 - figure supplement 3-1.tiff]

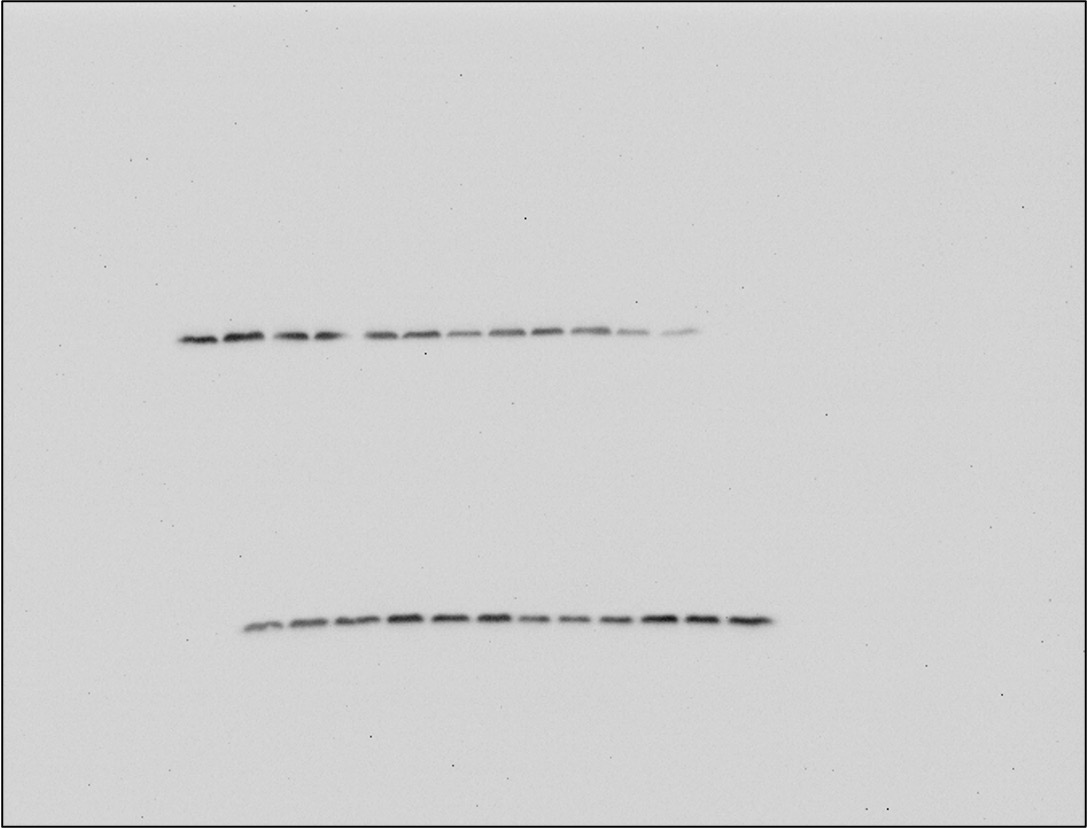

Supplement: Figure 2—source data 1. [file elife-86022-fig2-data1.zip › Figure 2 - source data 1/Figure 2 - figure supplement 4-2.jpg]

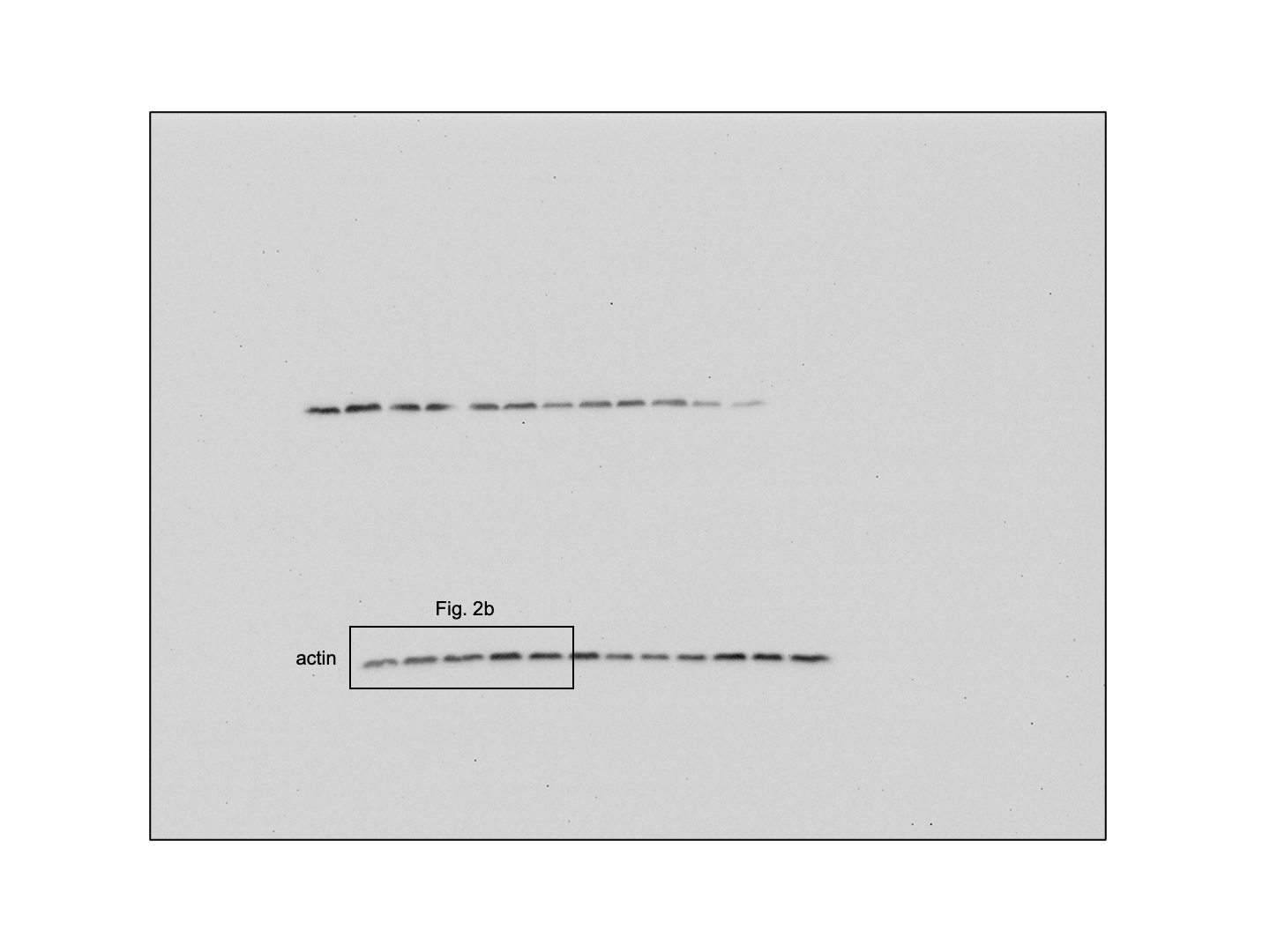

Supplement: Figure 2—source data 1. [file elife-86022-fig2-data1.zip › Figure 2 - source data 1/Figure 2 - figure supplement 4-1.tiff]

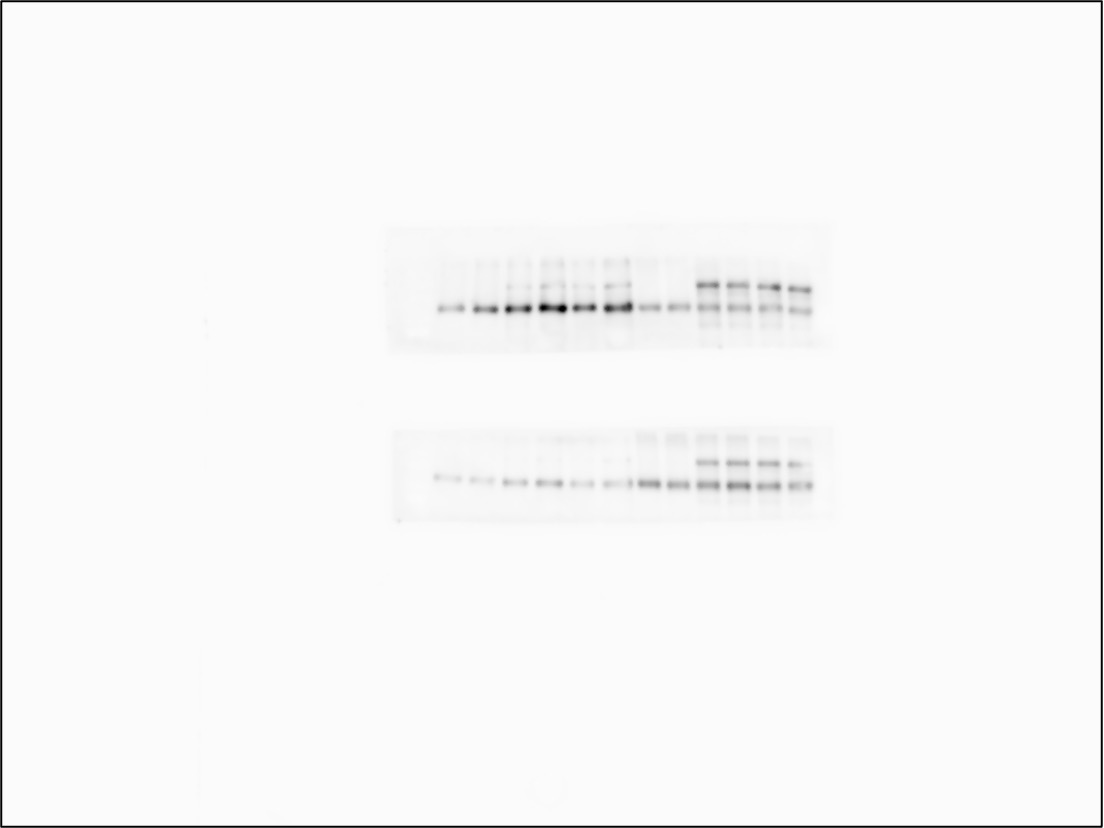

Supplement: Figure 2—source data 1. [file elife-86022-fig2-data1.zip › Figure 2 - source data 1/Figure 2 - figure supplement 3-2.jpg]

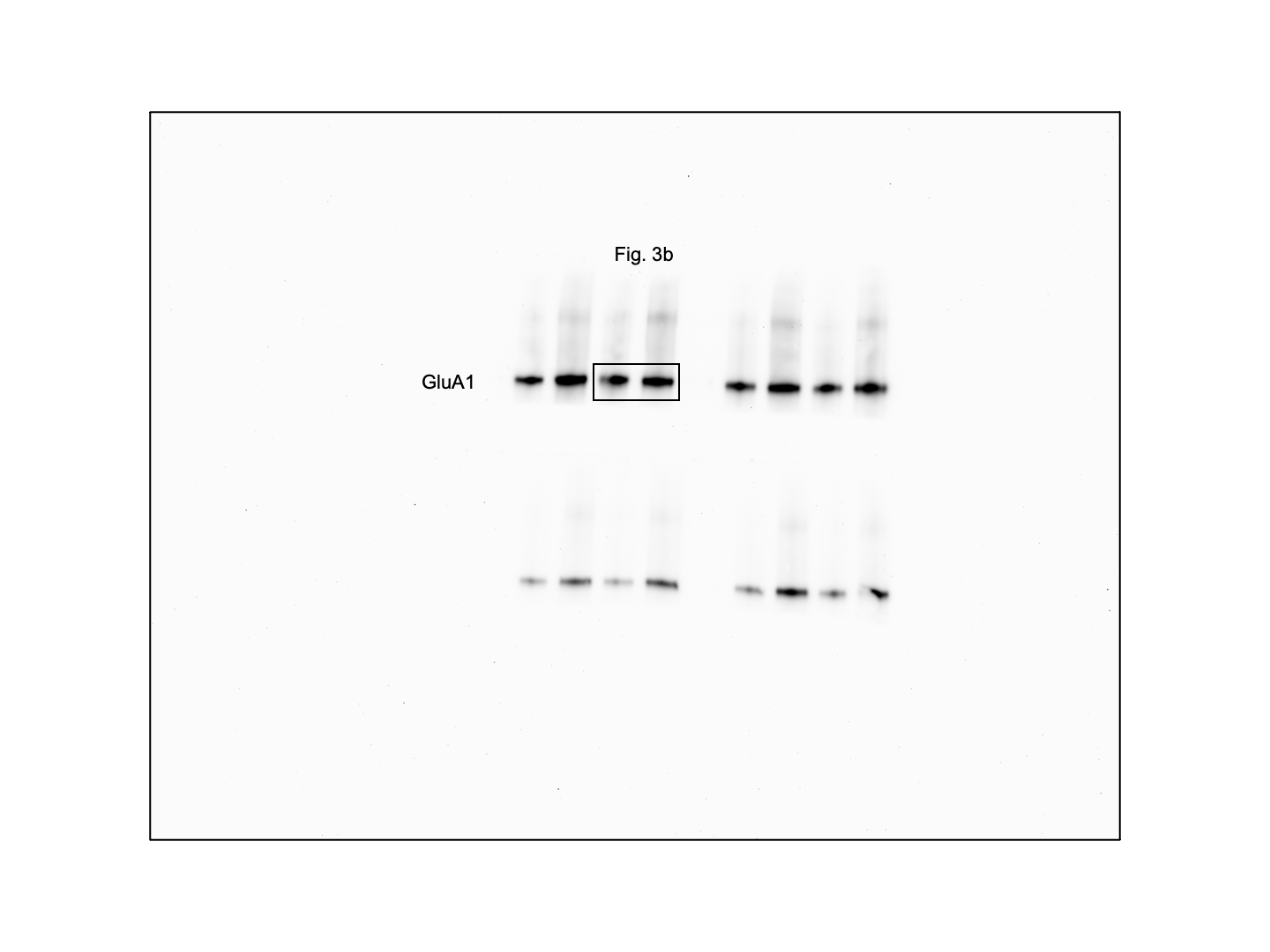

Supplement: Figure 3—source data 1. [file elife-86022-fig3-data1.zip › Figure 3 - source data 1/Figure 3 - figure supplement 6-1.tiff]

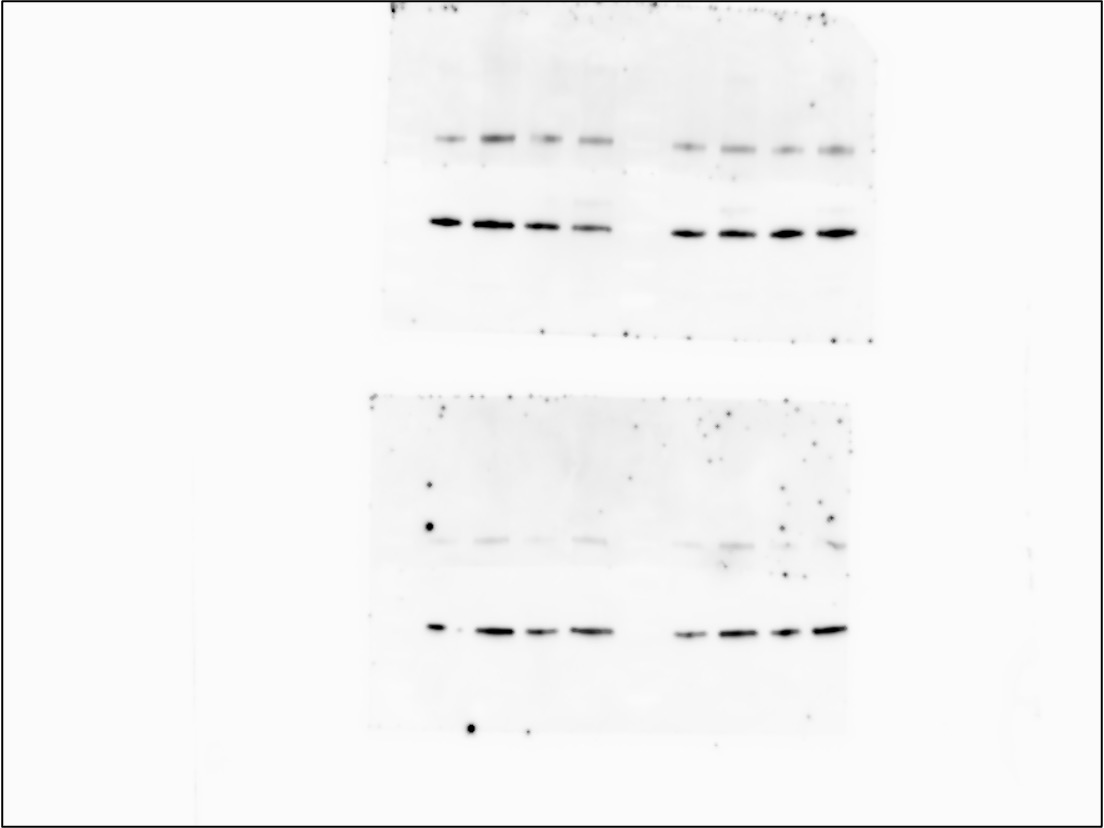

Supplement: Figure 3—source data 1. [file elife-86022-fig3-data1.zip › Figure 3 - source data 1/Figure 3 - figure supplement 8-2.jpg]

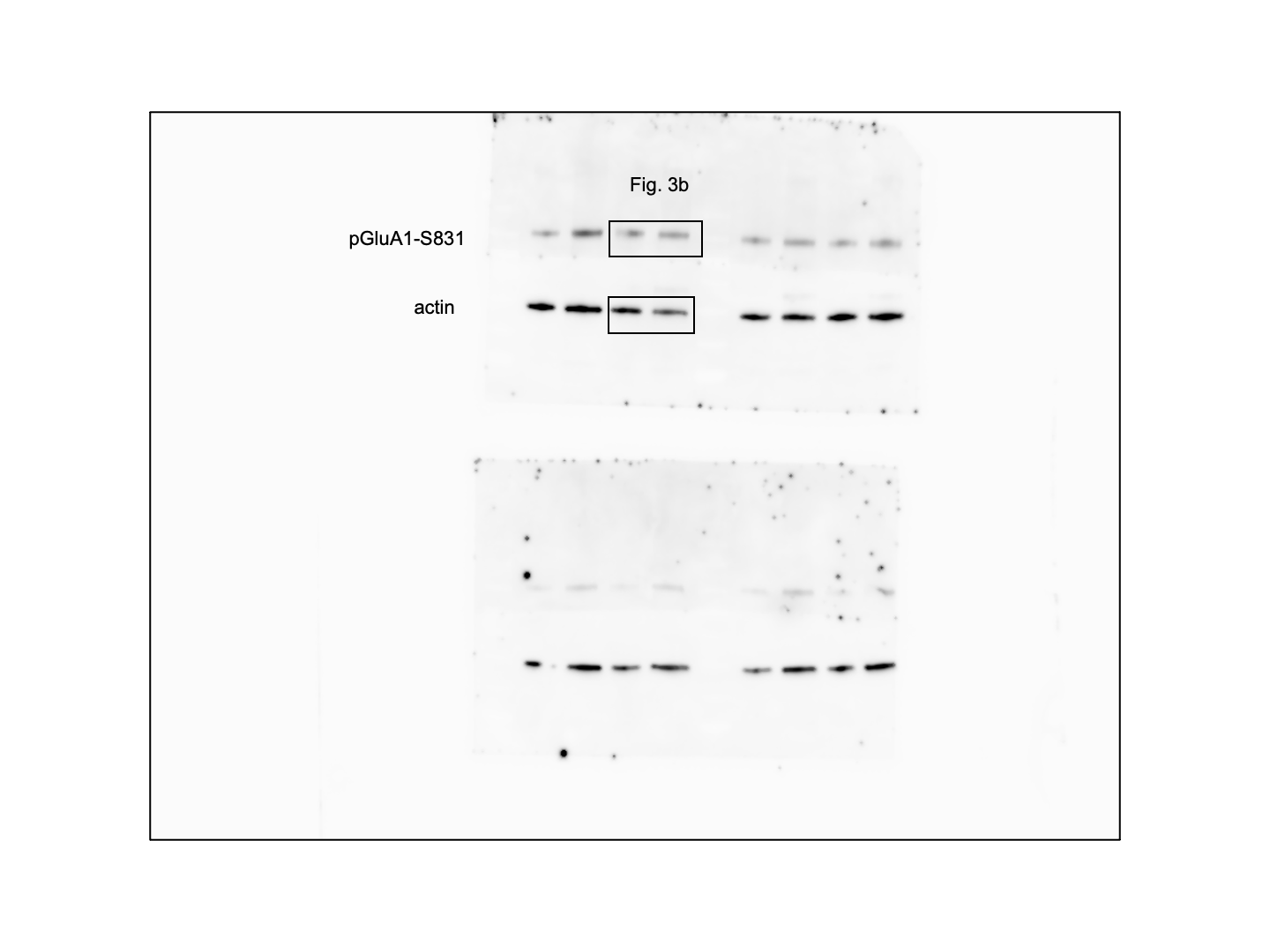

Supplement: Figure 3—source data 1. [file elife-86022-fig3-data1.zip › Figure 3 - source data 1/Figure 3 - figure supplement 8-1.tiff]

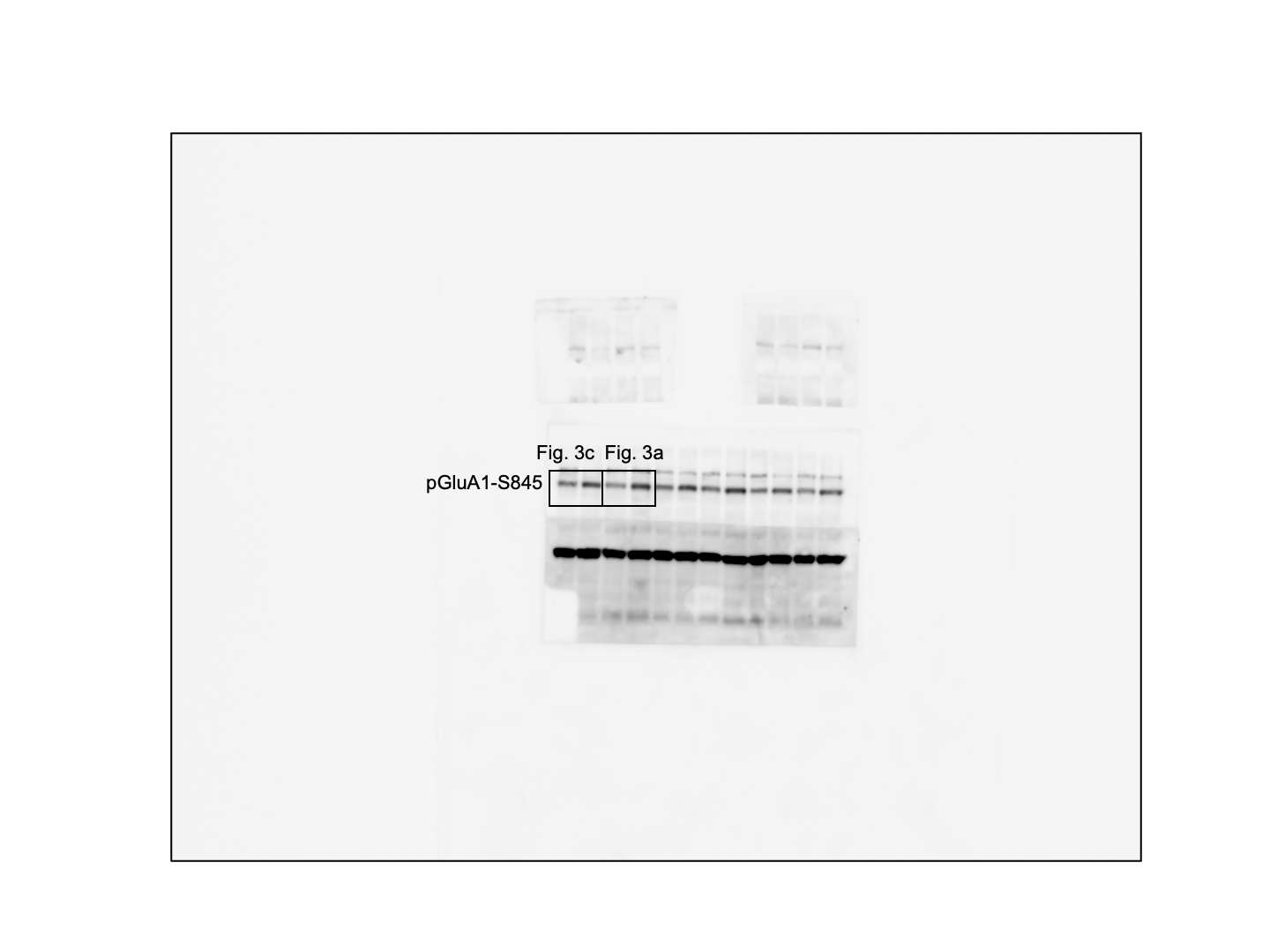

Supplement: Figure 3—source data 1. [file elife-86022-fig3-data1.zip › Figure 3 - source data 1/Figure 3 - figure supplement 4-1.tiff]

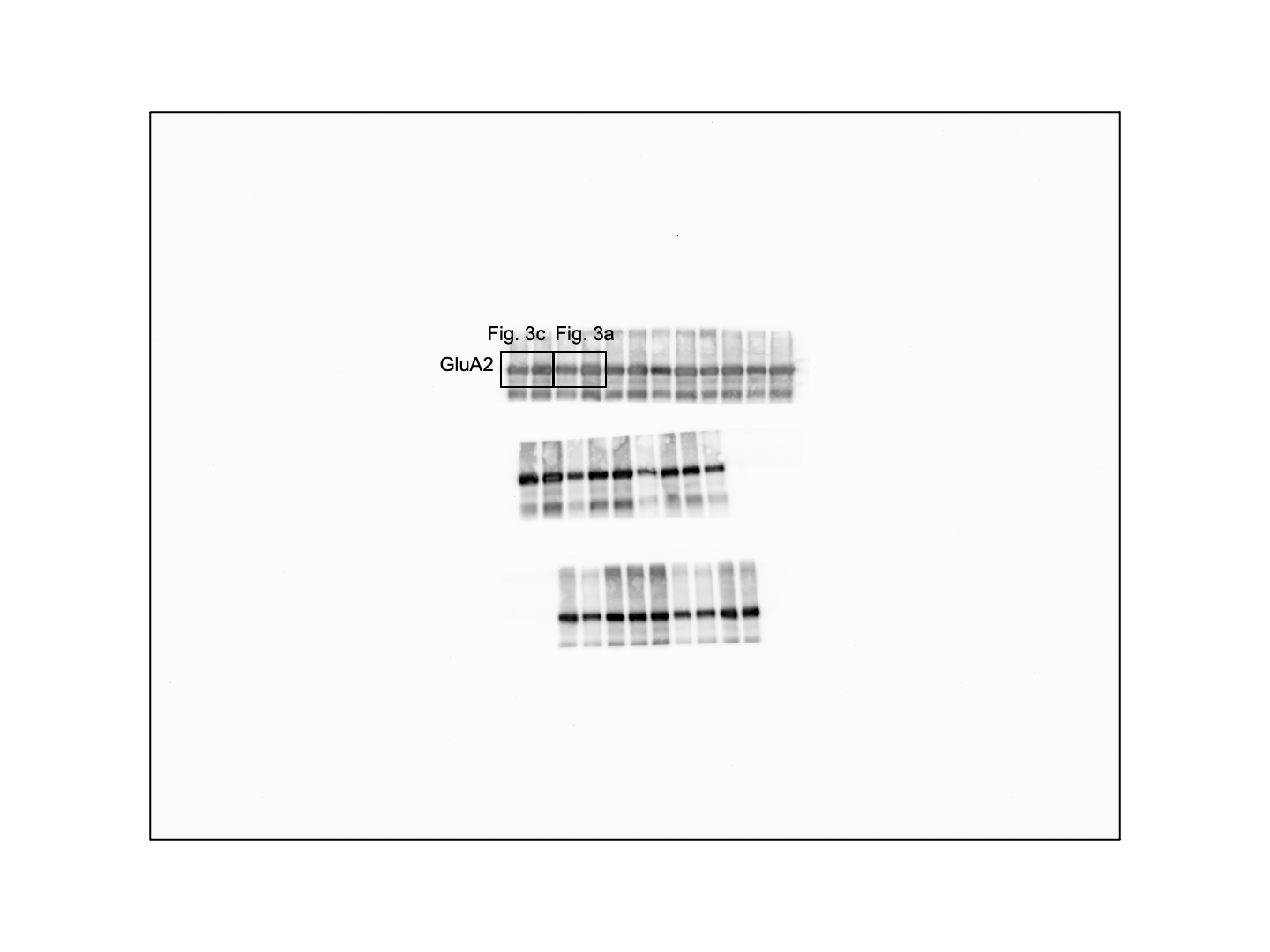

Supplement: Figure 3—source data 1. [file elife-86022-fig3-data1.zip › Figure 3 - source data 1/Figure 3 - figure supplement 2-1.tiff]

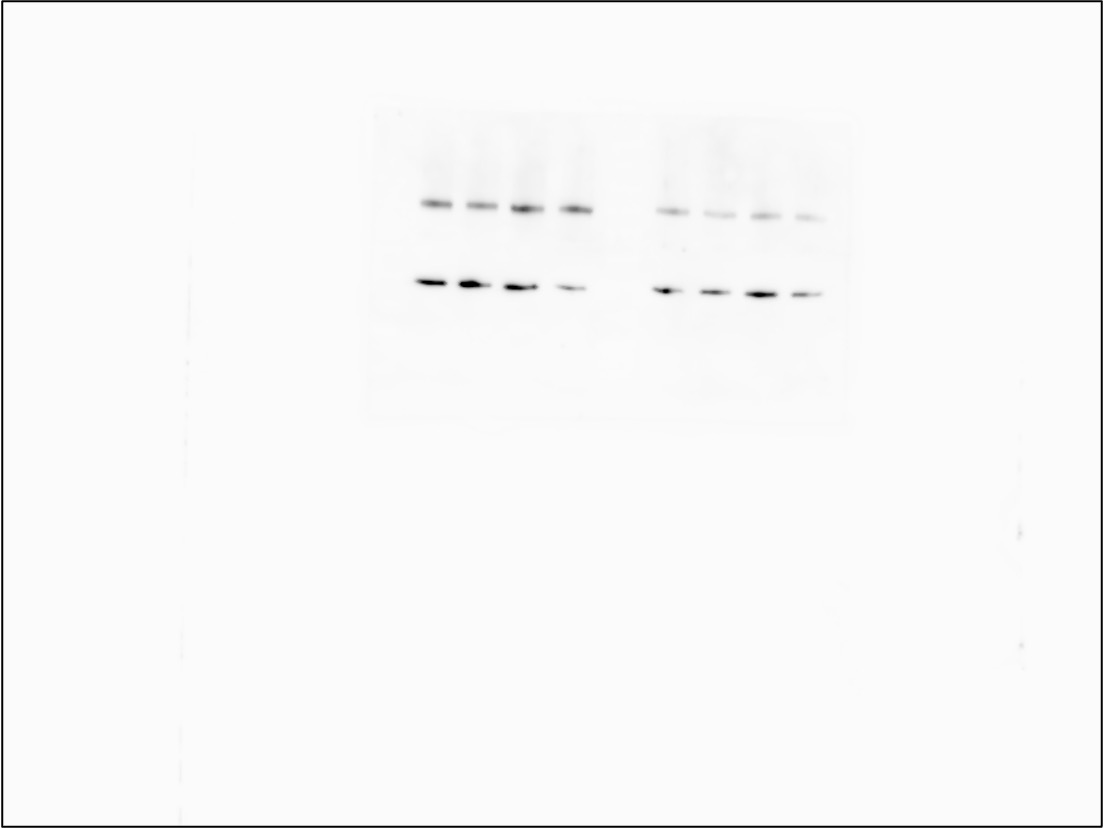

Supplement: Figure 3—source data 1. [file elife-86022-fig3-data1.zip › Figure 3 - source data 1/Figure 3 - figure supplement 9-2.jpg]

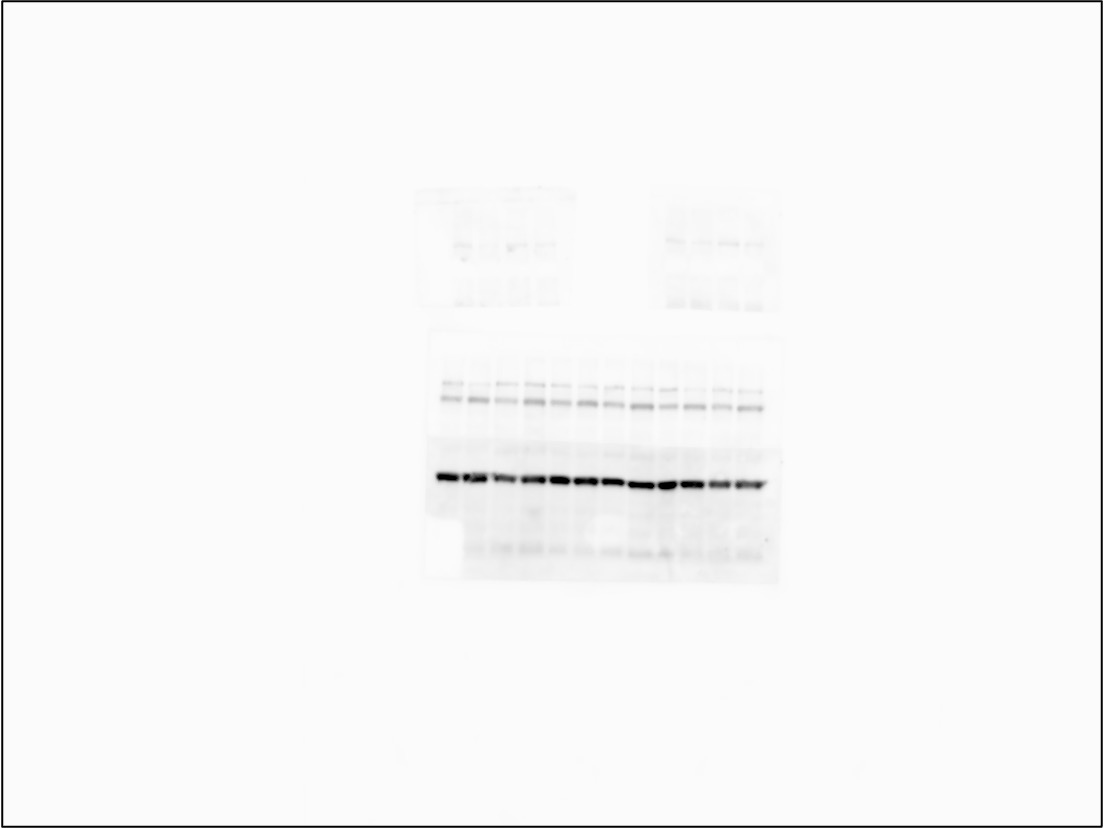

Supplement: Figure 3—source data 1. [file elife-86022-fig3-data1.zip › Figure 3 - source data 1/Figure 3 - figure supplement 5-2.jpg]

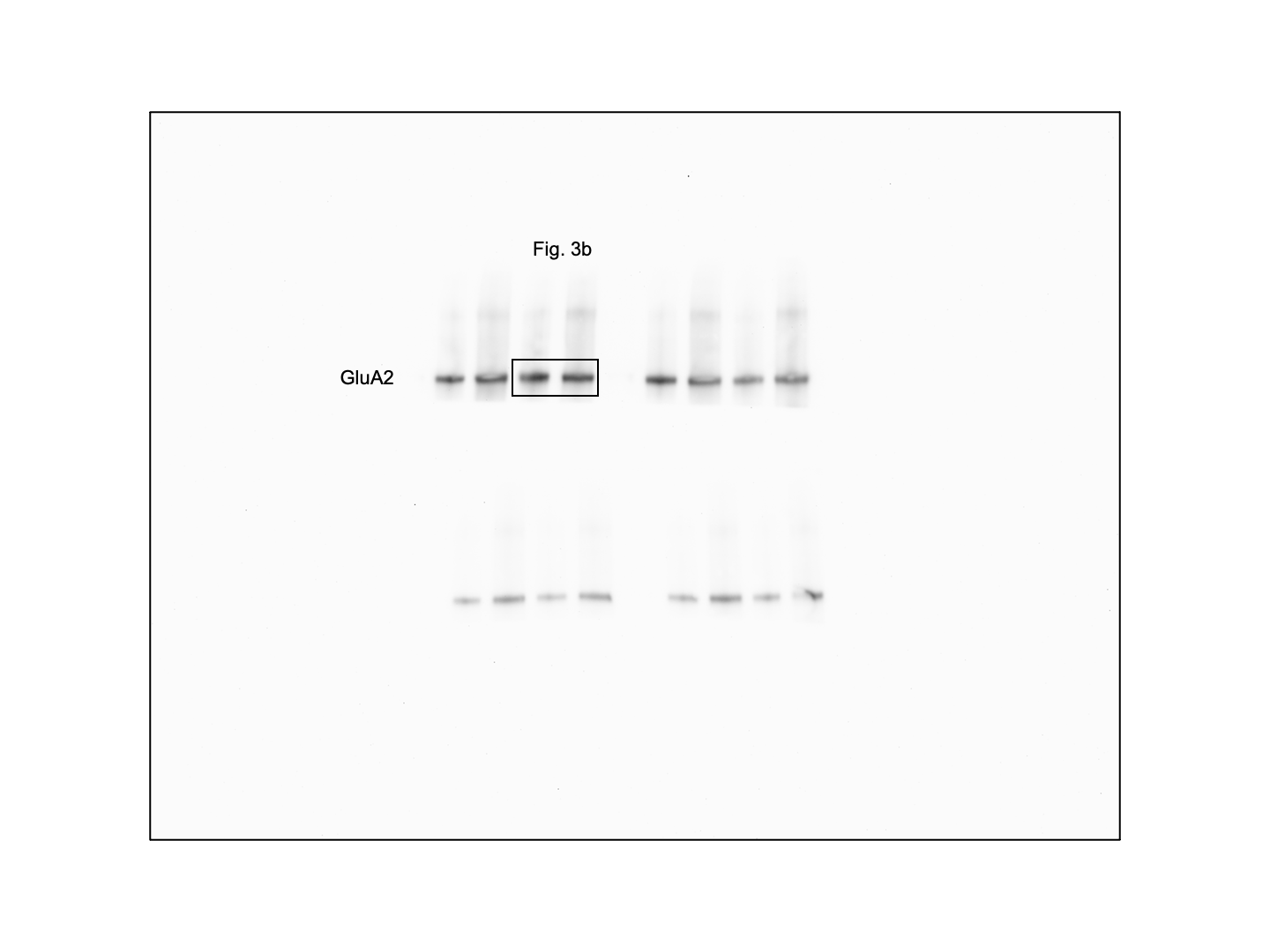

Supplement: Figure 3—source data 1. [file elife-86022-fig3-data1.zip › Figure 3 - source data 1/Figure 3 - figure supplement 7-1.tiff]

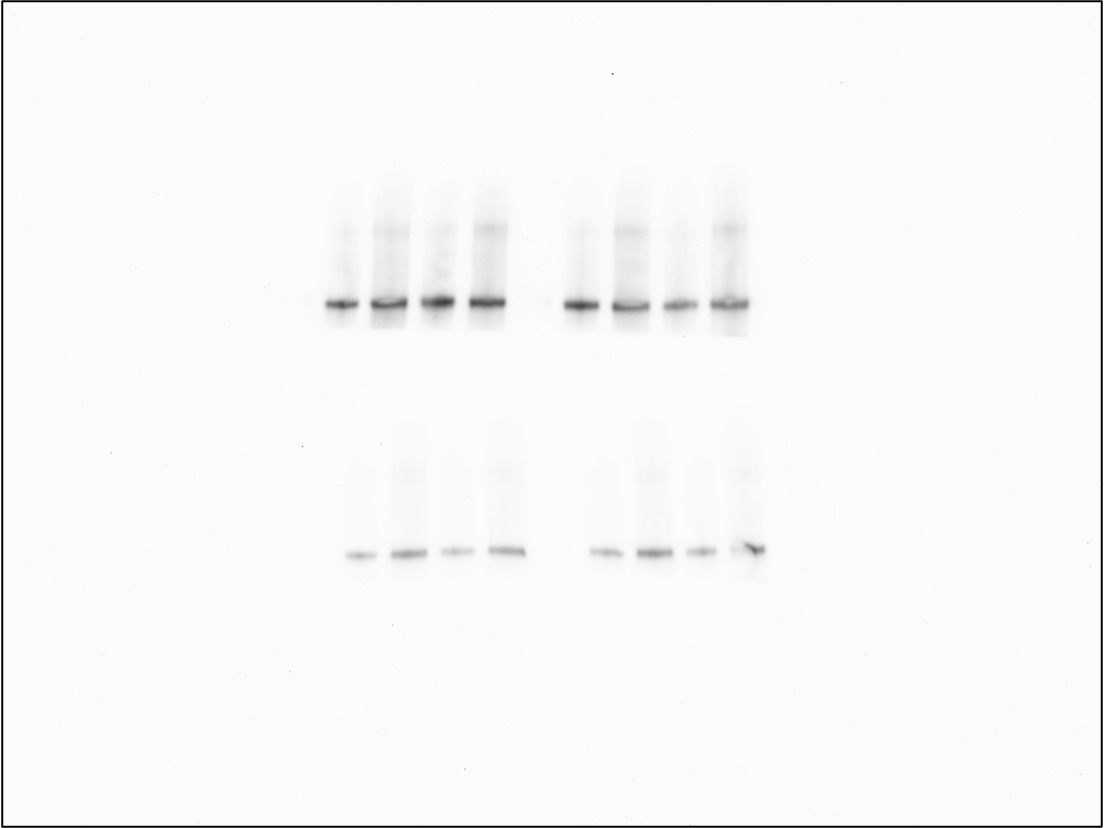

Supplement: Figure 3—source data 1. [file elife-86022-fig3-data1.zip › Figure 3 - source data 1/Figure 3 - figure supplement 7-2.jpg]

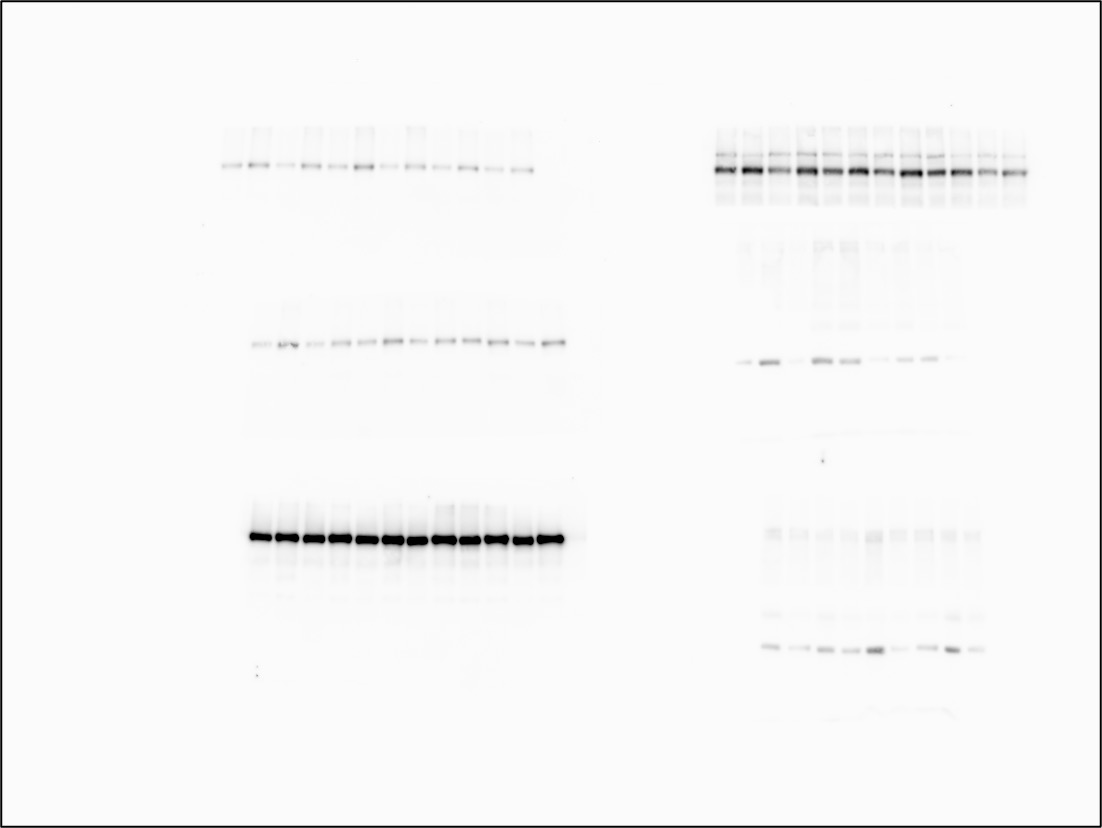

Supplement: Figure 3—source data 1. [file elife-86022-fig3-data1.zip › Figure 3 - source data 1/Figure 3 - figure supplement 3-2.jpg]

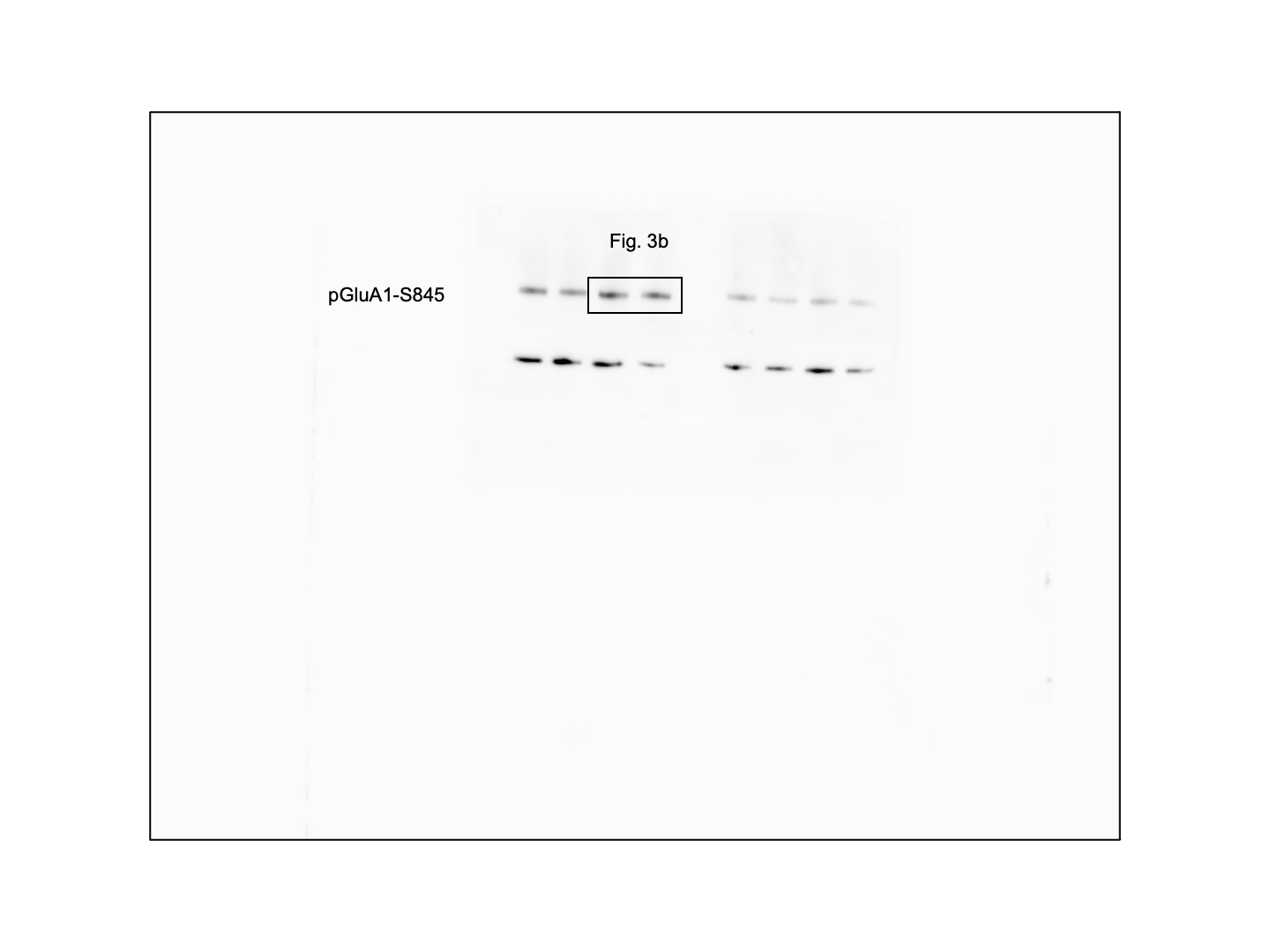

Supplement: Figure 3—source data 1. [file elife-86022-fig3-data1.zip › Figure 3 - source data 1/Figure 3 - figure supplement 9-1.tiff]

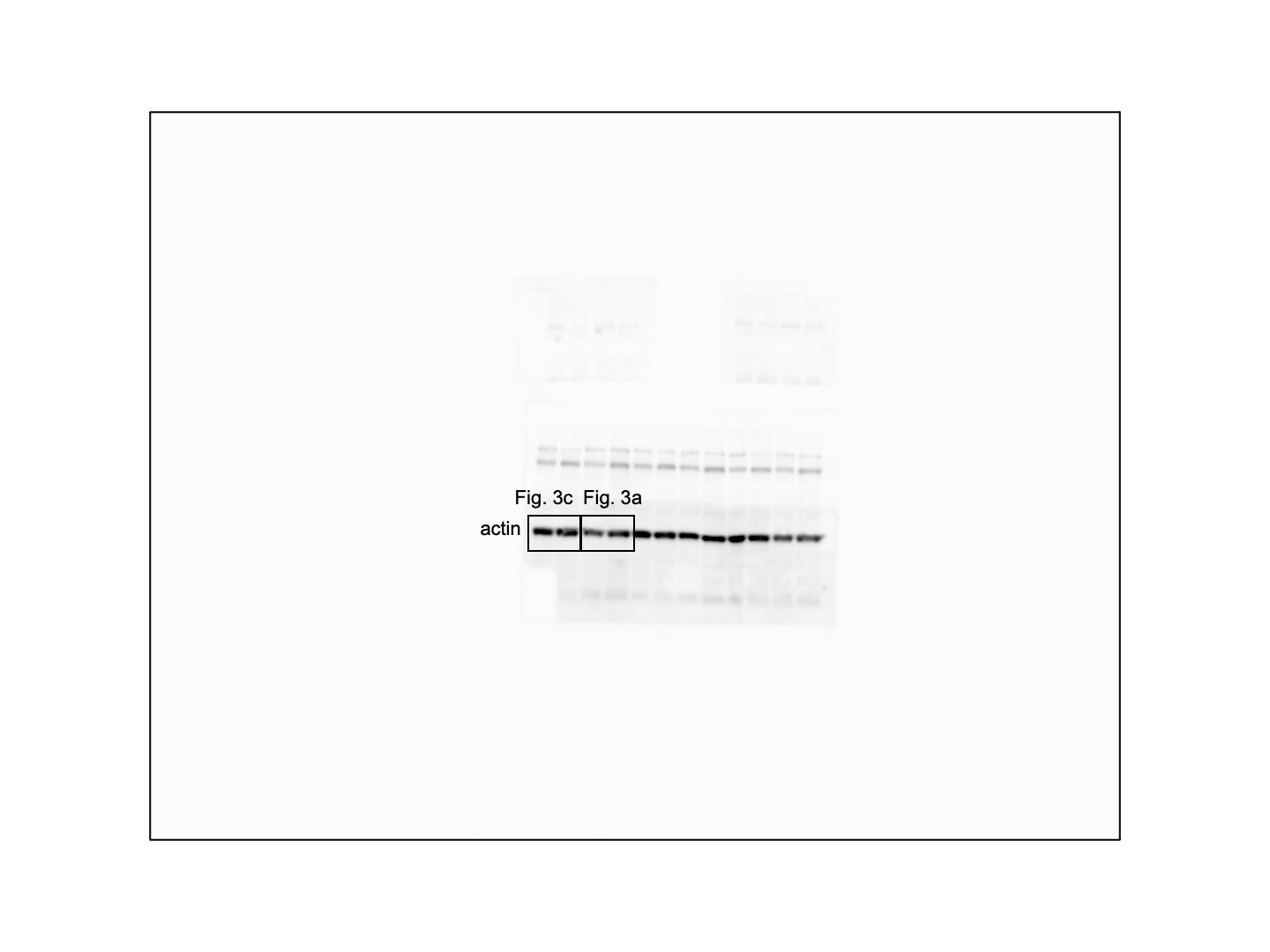

Supplement: Figure 3—source data 1. [file elife-86022-fig3-data1.zip › Figure 3 - source data 1/Figure 3 - figure supplement 5-1.tiff]

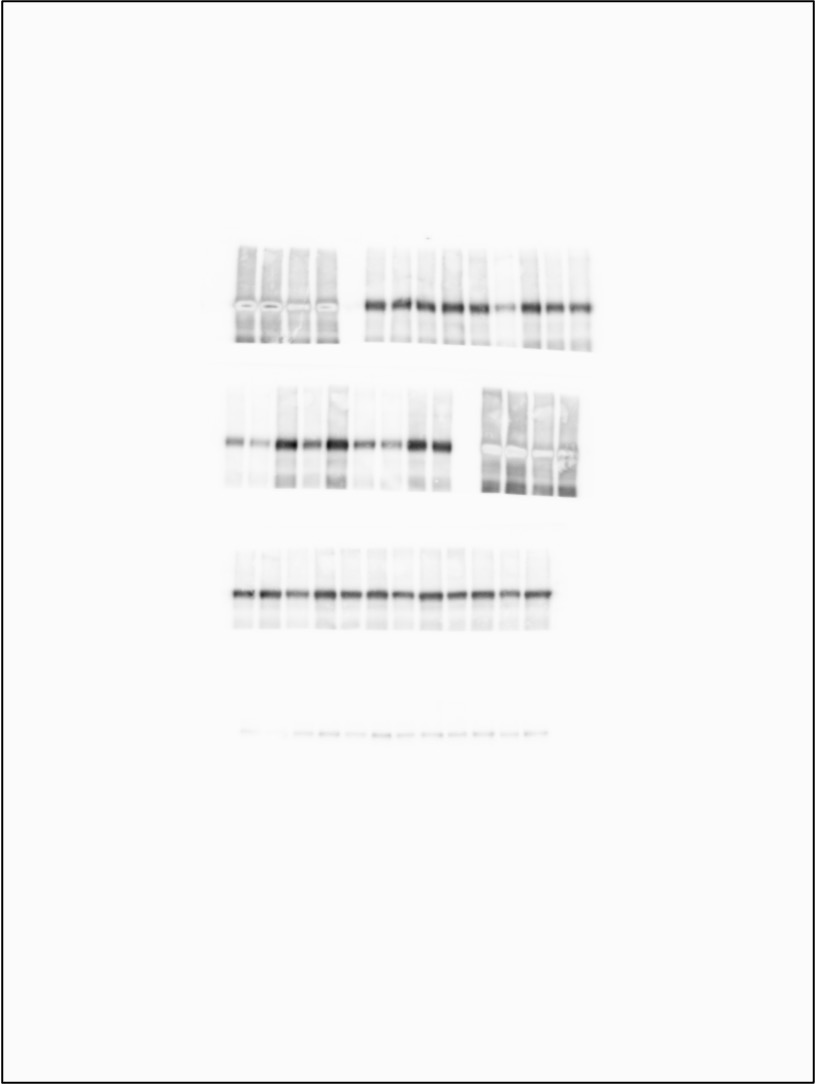

Supplement: Figure 3—source data 1. [file elife-86022-fig3-data1.zip › Figure 3 - source data 1/Figure 3 - figure supplement 1-2.jpg]

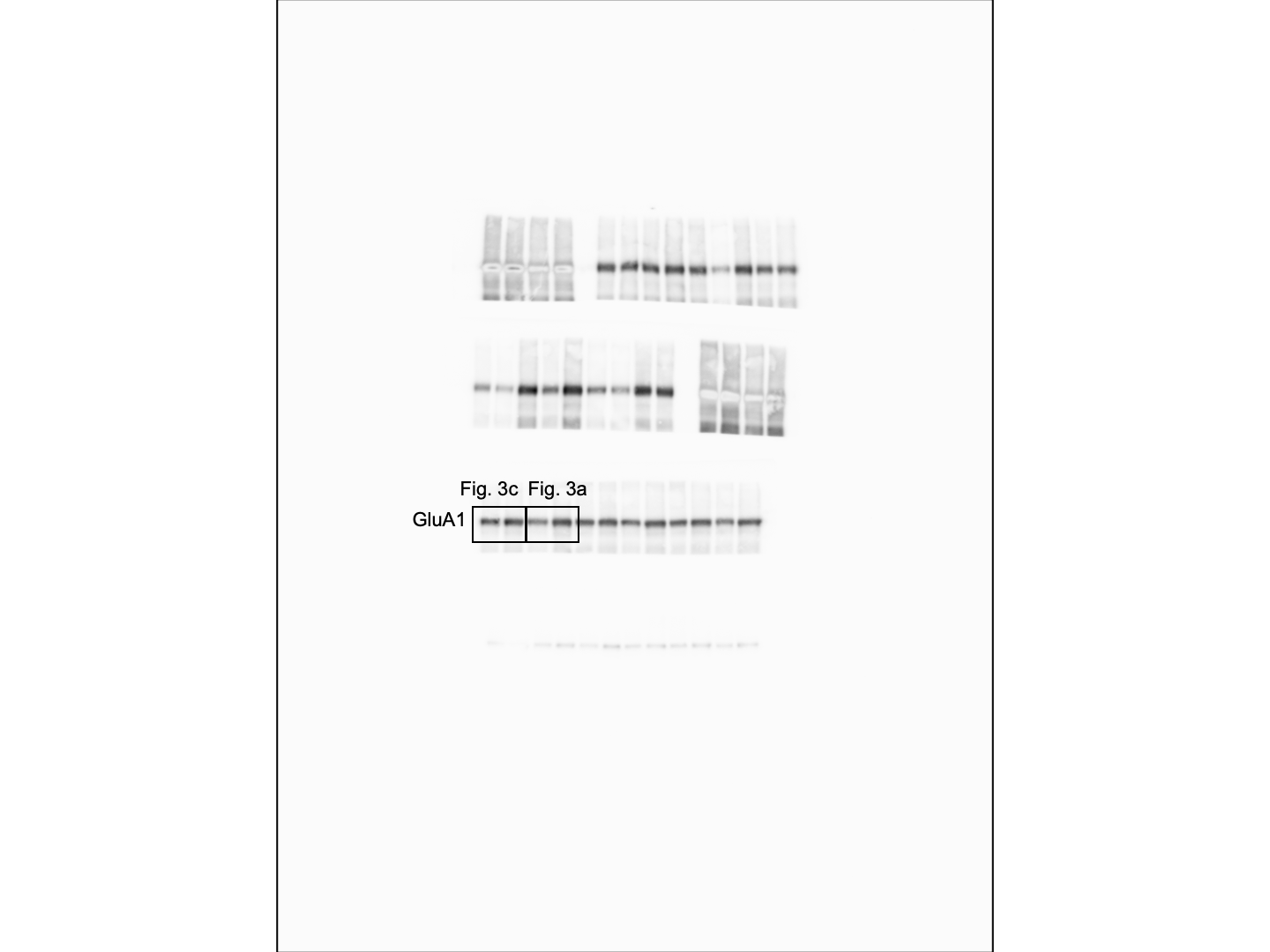

Supplement: Figure 3—source data 1. [file elife-86022-fig3-data1.zip › Figure 3 - source data 1/Figure 3 - figure supplement 1-1.tiff]

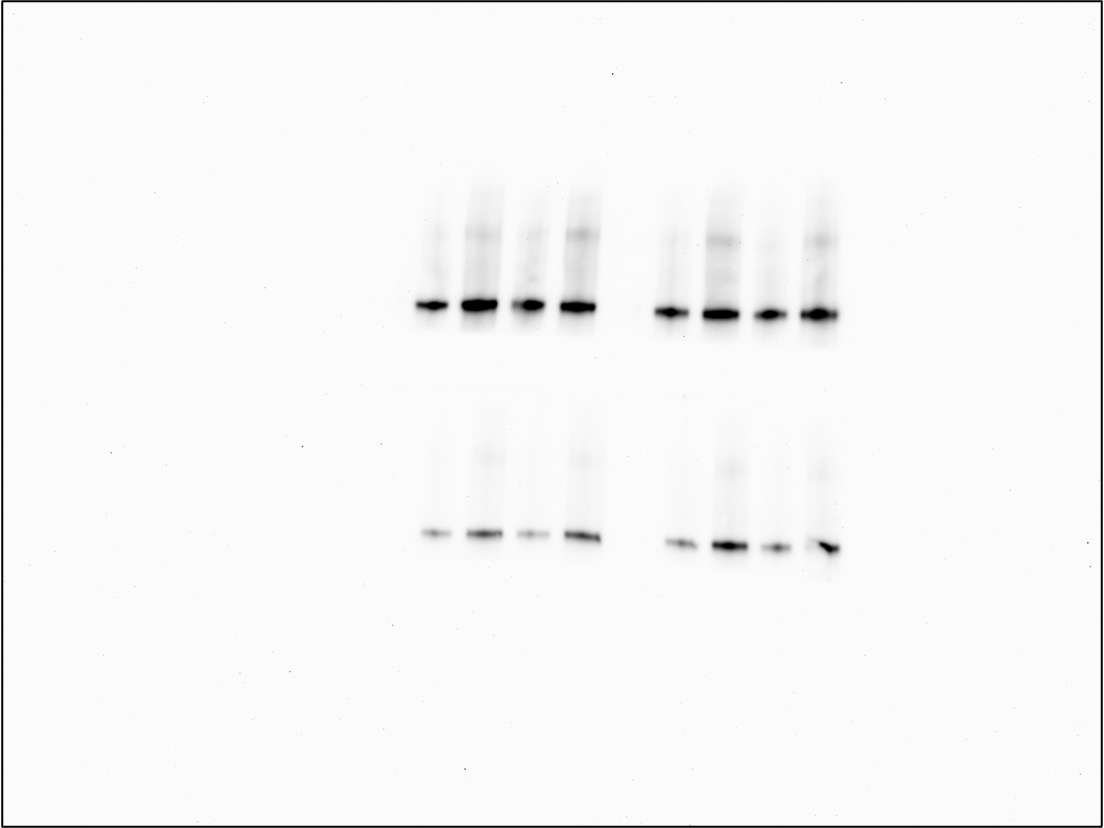

Supplement: Figure 3—source data 1. [file elife-86022-fig3-data1.zip › Figure 3 - source data 1/Figure 3 - figure supplement 6-2.jpg]

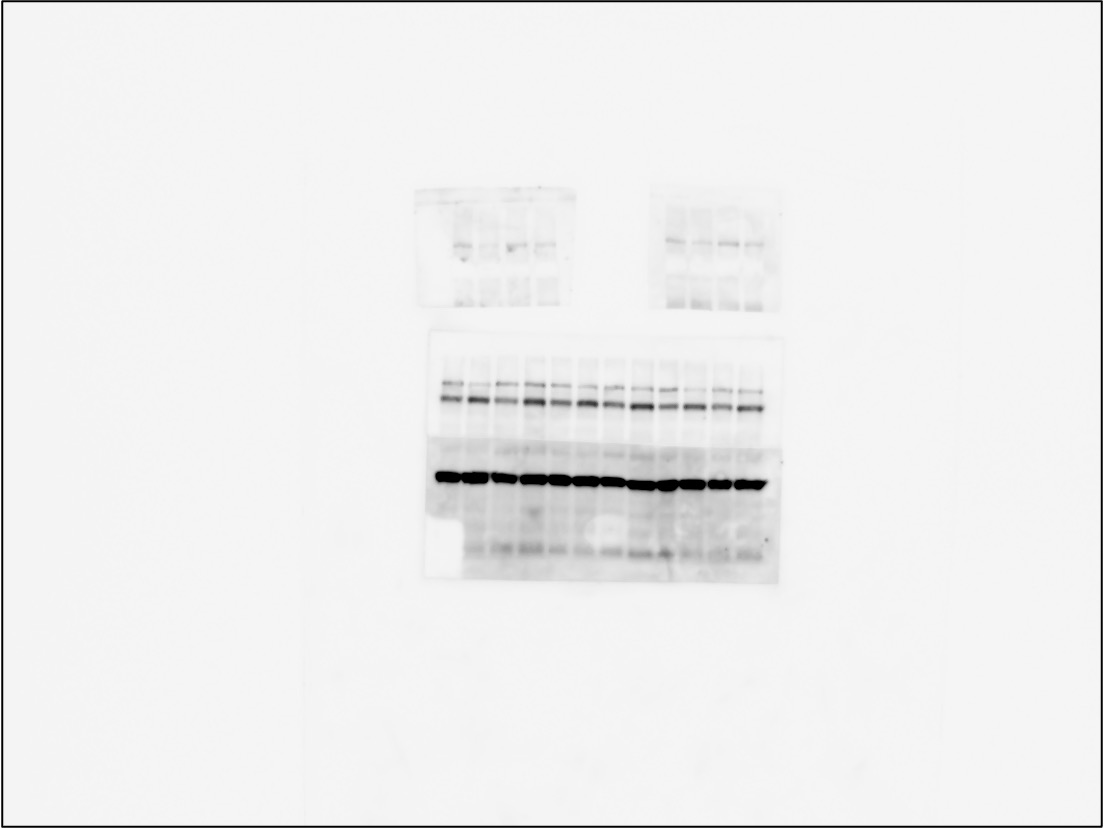

Supplement: Figure 3—source data 1. [file elife-86022-fig3-data1.zip › Figure 3 - source data 1/Figure 3 - figure supplement 4-2.jpg]

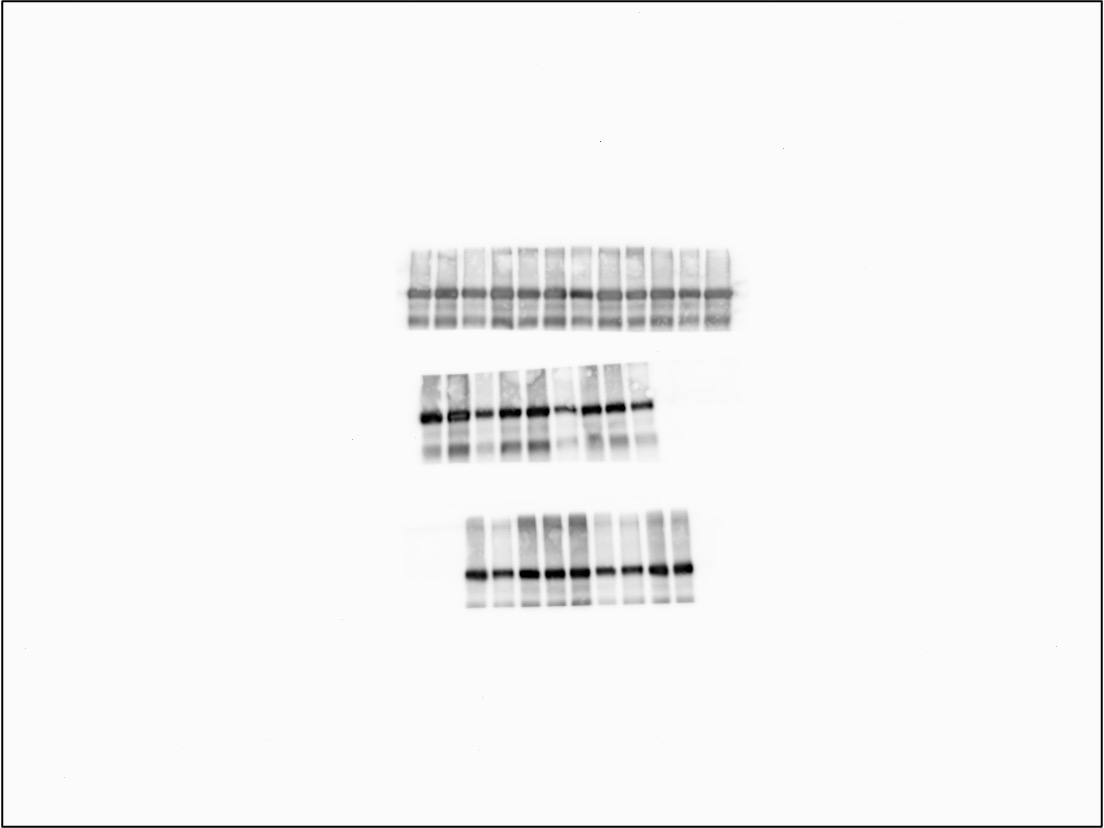

Supplement: Figure 3—source data 1. [file elife-86022-fig3-data1.zip › Figure 3 - source data 1/Figure 3 - figure supplement 2-2.jpg]

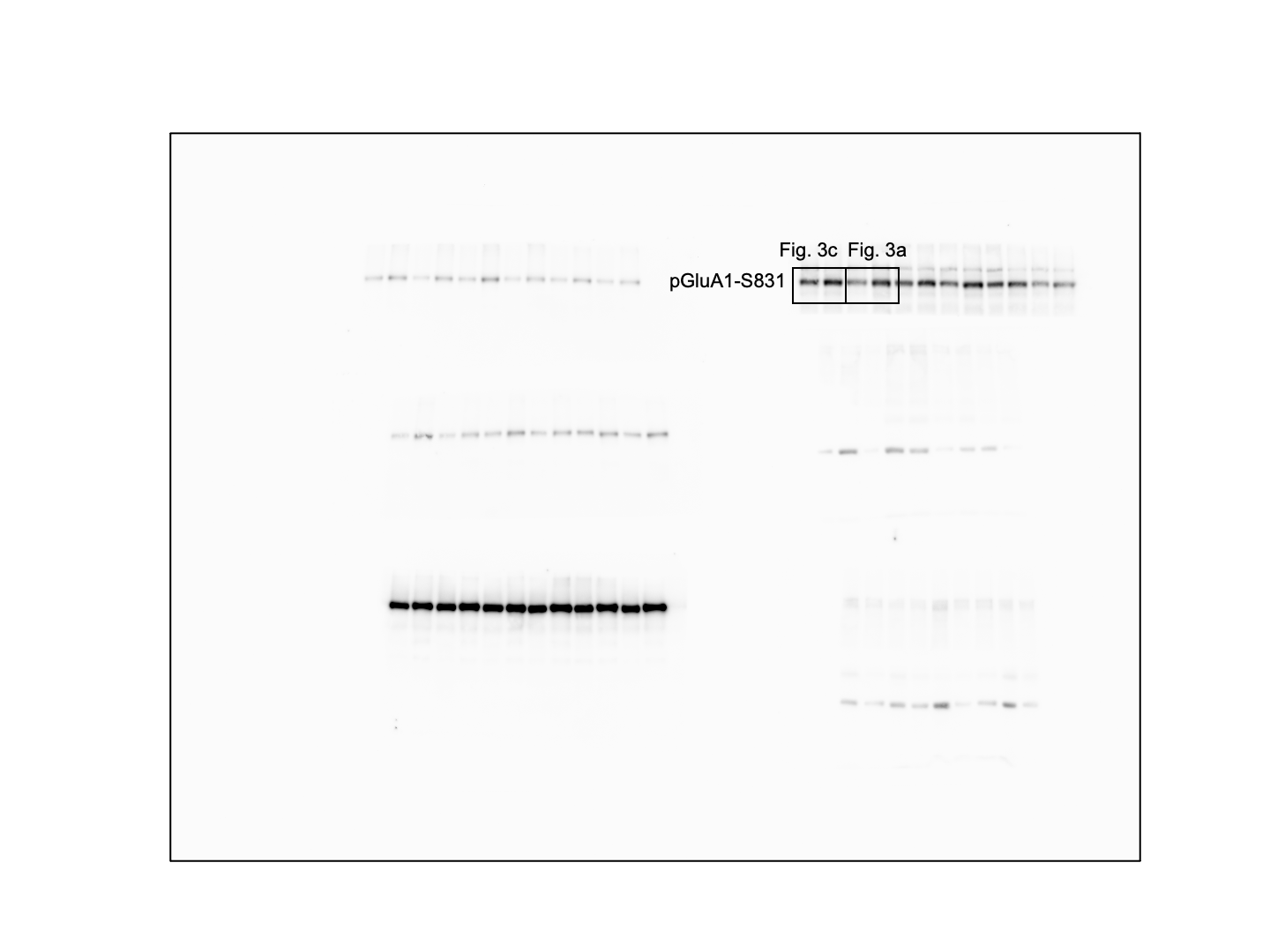

Supplement: Figure 3—source data 1. [file elife-86022-fig3-data1.zip › Figure 3 - source data 1/Figure 3 - figure supplement 3-1.tiff]
